# Evaluation of *Thiobacillus denitrificans*’ sustainability in nitrate-reducing Fe(II) oxidation and the potential significance of Fe(II) as a growth-supporting reductant

**DOI:** 10.1093/femsec/fiaf024

**Published:** 2025-03-17

**Authors:** Stefanie Becker, Thu Trang Dang, Ran Wei, Andreas Kappler

**Affiliations:** Geomicrobiology, Department of Geosciences, University of Tübingen, Schnarrenbergstrasse 94-96, 72076 Tübingen, Germany; Geomicrobiology, Department of Geosciences, University of Tübingen, Schnarrenbergstrasse 94-96, 72076 Tübingen, Germany; Institute for Modelling Hydraulic and Environmental Systems (IWS), Department of Stochastic Simulation and Safety Research for Hydrosystems, University of Stuttgart, Pfaffenwaldring 5a, 70569 Stuttgart, Germany; Geomicrobiology, Department of Geosciences, University of Tübingen, Schnarrenbergstrasse 94-96, 72076 Tübingen, Germany; Cluster of Excellence: EXC 2124: Controlling Microbes to Fight Infection, 72076 Tübingen, Germany

**Keywords:** *Thiobacillus denitrificans*, nitrate-reducing Fe(II) oxidation, nitrate-reducing Fe(II)-oxidizer, chemodenitrification

## Abstract

The betaproteobacterium *Thiobacillus denitrificans* (ATCC 25259) oxidizes Fe(II) while reducing nitrate, yet its capacity for autotrophic growth as a nitrate-reducing Fe(II)-oxidizer remains uncertain. This study explored this capacity through cultivation experiments across multiple transfers, using growth medium with Fe(II) and nitrate as sole electron donor and acceptor, respectively. This setup necessitated nitrate reduction coupled to Fe(II) oxidation as the primary energy-yielding mechanism and Fe(II) as the exclusive electron donor for CO_2_ fixation and biomass production. Thiosulfate/nitrate pregrown *T. denitrificans* oxidized 42% of 10 mM Fe(II), reduced 54% of 3.5 mM nitrate, and accumulated 1.6 mM nitrite, but showed no cell growth. Subsequent transfers from this Fe(II)/nitrate culture to fresh medium with Fe(II) and nitrate showed no nitrate-reducing Fe(II) oxidation or population growth. While bacterial activity [Fe(II) oxidation, nitrate reduction] occurred in the first transfer from thiosulfate/nitrate to Fe(II)/nitrate, nitrite was produced, further reacting with Fe(II) abiotically (chemodenitrification). A kinetic model assessed enzymatic versus abiotic Fe(II) oxidation, revealing enzymatic oxidation accounted for twice as much (ca. 70%) as abiotic denitrification (ca. 30%) within 22 days. These findings suggest *T. denitrificans* performs the first step of denitrification with Fe(II) as an electron donor but does not achieve autotrophic growth under these conditions.

## Teaser

*Thiobacillus denitrificans* does not grow chemolithoautotrophically under nitrate-reducing, Fe(II)-oxidizing conditions and cannot sustain nitrate-reducing Fe(II) oxidation over multiple transfers, and was therefore classified as a non-autotrophic nitrate-reducing Fe(II)-oxidizer.

## Introduction

The prevalence of nitrate pollution from agriculture, coupled with widespread iron occurrence in the environment (OECD 2008, Rivett et al. 2008), highlights the potential importance of denitrifying iron(II)-oxidizers in the environment (Kappler et al. 2021) and in wastewater treatment, offering potential ecofriendly solutions for remediating nitrate-polluted groundwater (Zhang et al. 2016, 2018, Jakus et al. 2021, Jokai et al. 2021, Grimm et al. 2024). These bacteria, collectively termed nitrate-dependent iron(II)-oxidizing bacteria (NDFeO), play a crucial role in merging the biogeochemical iron and nitrate cycles (Kappler et al. 2021). In the literature the term NDFeO is used for both, bacteria that catalyze Fe(II) oxidation enzymatically, and heterotrophic denitrifiers, which oxidize Fe(II) indirectly via reactive nitrogen species (N-species), intermediates of denitrification (Sørensen and Thorling 1991, Tai and Dempsey 2009). In the case of enzymatic Fe(II) oxidation, it is biochemically coupled to nitrate reduction, leading to the classification of related bacteria as nitrate-reducing Fe(II)-oxidizers (NRFeOx). This subgroup can be further categorized into (1) bacteria with a carbon demand that relies on organic carbon, such as chemolithoheterotrophs (mixotrophic NRFeOx), and (2) those that are chemolithoautotrophs (autotrophic NRFeOx). While studying bacterial communities remains challenging, working with pure bacterial strains, especially colony-forming cells, is more straightforward. Molecular biology tools developed for these strains allow for detailed assessment and a deeper understanding of their metabolic mechanisms. Thus, the identification of a model organism as single bacterial strain for studying autotrophic NRFeOx is essential. For example, cultures KS, BP, and AG have been used as model enrichment cultures to study chemolithoautotrophic NRFeOx instead of a pure culture (Huang et al. 2021a, b, Jakus et al. 2021). Despite several individual pure strains showing promise [reviewed by Bryce et al. (2018)], none have been sufficiently studied to be classified as chemolithoautotrophic NRFeOx (Bryce et al. 2018). Potential candidates should (1) not require any organic carbon to meet their whole carbon demand, (2) show cell growth with only Fe(II), nitrate and CO_2_ as energy and growth substrates, (3) maintain Fe(II) oxidation over several transfers under autotrophic conditions, and (4) incorporate labelled CO_2_ into biomass during Fe(II) oxidation (Bryce et al. 2018).

Identifying an organism meeting all criteria is crucial for unraveling the molecular mechanisms underlying chemolithoautotrophic nitrate reduction coupled to iron(II) oxidation. As the process remains incompletely elucidated, it lacks a formal designation. For clarity and consistency with Becker et al. (2021), this process will be referred to as chemolithoautotrophic nitrate-reducing Fe(II) oxidation (Becker et al. 2021). In the present study, we selected *Thiobacillus denitrificans* (ATCC 25259) as a potential autotrophic NRFeOx by confirming criteria 1–3. *Thiobacillus denitrificans* (*T. denitrificans*) was suggested to be an obligate chemolithoautotrophic, facultative anaerobic, Gram-negative bacterium known to couple denitrification to sulfur, uranium, and Fe(II) oxidation (Beijerinck 1904, Straub et al. 1996, Kelly and Wood 2000, Beller 2005, Kelly et al. 2005, Beller et al. 2006a). Given the suite of molecular tools available for *T. denitrificans* (single colony growth, microarray analysis, targeted gene knockouts, and random transposon mutagenesis), this isolate might represent an ideal chemolithoautotrophic NRFeOx model organism (Beller et al. 2006b, 2013, Letain et al. 2007). Enzymatic Fe(II) oxidation was initially reported for *T. denitrificans* by Straub et al. (1996), whereas Beller et al. (2013) demonstrated a strong correlation between Fe(II) oxidation and nitrate reduction via a whole-cell suspension assay and conducted cultivation experiments to evaluate autotrophic growth (Straub et al. 1996, Beller et al. 2013).

However, despite extensive studies, understanding the Fe(II) oxidation mechanism of *T. denitrificans* remains challenging (Beller et al. 2013). While *T. denitrificans* stands out as one of the most extensively studied potential autotrophic NRFeOx, there still exists a deficiency in data supporting sustained cell growth under nitrate-reducing, Fe(II)-oxidizing conditions.

This article presents an investigation into whether *T. denitrificans* can proliferate through energy conservation from nitrate-reducing Fe(II) oxidation and utilize Fe(II) as the sole growth-supporting electron donor for CO_2_ fixation. To address this, we established a novel protocol for direct cell counting of Fe(II)-oxidizers using flow cytometry. Cultures of *T. denitrificans* were monitored over a 22-day period in medium containing nitrate and Fe(II), with transfers to fresh medium to assess its ability to persist and proliferate under nitrate-reducing Fe(II)-oxidizing conditions. Various controls were employed to exclude heterotrophic or thiosulfate-induced growth, ensuring the observed effects were attributable to nitrate-reducing Fe(II) oxidation.

## Materials and methods

### Source of microorganism

*Thiobacillus denitrificans* strain ATCC 25259, originally isolated by B. F. Taylor in the 1960s, was obtained from the American Type Culture Collection (ATCC), where it was freeze-dried for storage and distribution (Taylor and Hoare 1971, Taylor et al. 1971). *Thiobacillus denitrificans* is maintained in our laboratory since 2019 using thiosulfate as electron donor under denitrifying conditions, as described elsewhere (DSMZ 2024).

### Media preparation and experimental setup

For the preculture and positive growth control, thiosulfate-containing medium was prepared, as described by Leibniz Institute DSMZ (German Collection of Microorganisms and Cell Cultures), containing 12 mM bicarbonate, 20 mM thiosulfate, and 20 mM nitrate. 50 ml of medium were distributed into 100 ml serum bottles and 25 ml of medium into 50 ml serum bottles for precultures or positive growth controls, respectively (DSMZ 2024). All cultures were incubated at 30°C.

**Figure 1.**
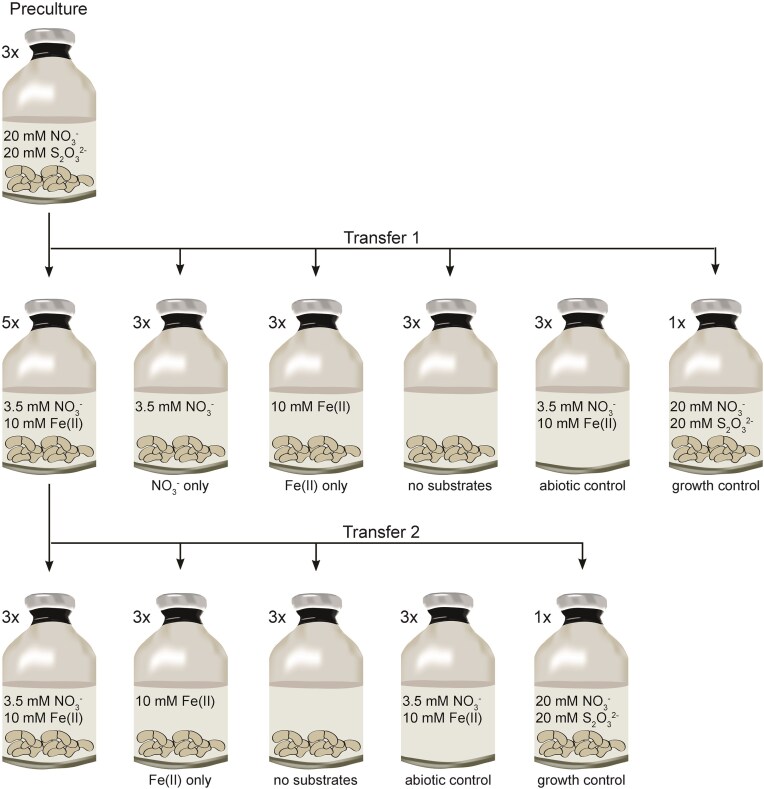
Overview of the experimental setup. First, a preculture was grown under denitrifying conditions with thiosulfate as electron donor in 50 ml medium, containing 12 mM bicarbonate, 20 mM thiosulfate, and 20 mM nitrate (DSMZ 2024). In the late exponential growth phase, the cells were harvested by centrifugation, washed, and inoculated (Transfer 1) into five bottles with 50 ml of medium each containing 30 mM bicarbonate, 10 mM Fe(II), and 3.5 mM nitrate and into three bottles with nitrate only as well as three bottles with Fe(II) only. The final cell concentration was ${\mathrm{4 \times 1}}{{\mathrm{0}}^{\mathrm{7}}}$ cells/ml after inoculation. In addition, three bottles with cells only (no substrates), an abiotic control [with Fe(II) and nitrate], and a positive growth control (thiosulfate/nitrate) were set up. Finally, the cell/mineral aggregates from the Fe(II)-oxidizing denitrifying culture from Transfer 1 were washed and transferred (Transfer 2) to fresh medium containing 10 mM Fe(II) and 3.5 mM nitrate, to medium lacking nitrate or lacking both Fe(II) and nitrate, and finally into medium containing thiosulfate and nitrate, serving as a growth control. The final cell concentration was ${\mathrm{3 \times 1}}{{\mathrm{0}}^{\mathrm{6}}}$ cells/ml after inoculation.

Fe(II) oxidation, nitrate reduction, and growth by *T. denitrificans* were evaluated under a 90% N_2_/10% CO_2_ atmosphere at 30°C using basal freshwater medium as described by Widdel and Bak (1992) amended with 10 mM FeSO_4_ and 3.5 mM NaNO_3_ as described by Straub et al. (1996) (Widdel and Bak 1992). The basal medium was buffered by 30 mM bicarbonate while being exposed to 10% CO_2_ atmosphere and the pH was adjusted to 7.2 using 1 M HCl. The medium was distributed to Schott-bottles using a Widdel-flask. FeSO_4_ and NaNO_3_ were added using syringes, according to the different setups as shown in Fig. 1. 50 ml of medium were then distributed into 100 ml serum bottles. The initial overpressure of the serum bottles has been adjusted to atmospheric pressure using a gas trap, prior to inoculation with bacteria. The inoculum for the first transfer was 10% (v/v) of a thiosulfate-/nitrate-grown culture harvested in late exponential phase (${\mathrm{4 \times 1}}{{\mathrm{0}}^{\mathrm{7}}}$ cells/ml final concentration). The cells were washed twice with basal medium lacking FeSO_4_ and NaNO_3_^−^, by centrifugation (10 min, 6200 × *g* and 20°C). The biological replicates were tested along with controls lacking either FeSO_4_ or NaNO_3_^−^, or lacking both as shown in Fig. 1. The Fe(II)-oxidizing denitrifying setup was carried out in five biological replicates, negative controls in triplicates and the positive growth control (thiosulfate/nitrate) once. After 22 days, the Fe(II)-oxidizing denitrifying cultures were harvested, washed (10 min, 6200 × *g* and 20°C) and used as inoculum for the second transfer. All harvested cells were transferred (${\mathrm{3 \times 1}}{{\mathrm{0}}^{\mathrm{6}}}$ cells/ml final concentration for all setups) to triplicates containing both 10 mM FeSO_4_ and 3.5 mM NaNO_3_^−^, only FeSO_4_ and lacking both substrates as shown in Fig. 1.

While the harvested cells for the first transfer were iron free (since they were pregrown with thiosulfate), the inoculum for the second transfer contained some Fe(II) minerals (such as vivianite and siderite) that precipitated in the cultures of the first transfer with the Fe(II) added and the phosphate/bicarbonate in the growth medium, and some Fe(III) minerals that formed during Fe(II) oxidation in the first transfer. Consequently, a no-Fe(II) control was impossible for the second transfer. Nitrogen and iron speciation (nitrate, nitrite, ammonia, ferrous, and ferric iron), and cell numbers were quantified by photometric measurements using flow injection analysis, colorimetric ferrozine-based assay, and flow cytometry, respectively, and the cell viability was evaluated by dead/live staining and fluorescence microscopy as described below.

### Sample collection

Samples were withdrawn from the bottles anoxically with a syringe through a rubber stopper, while gently mixed by hand, inside of an anoxic glovebox. The samples were used for cell counts and quantification of Fe(tot) and Fe(II) [the sum of dissolved and solid phase Fe(tot) and Fe(II), respectively], whereas the remaining sample was centrifuged for 5 min at 12 408 × *g* and room temperature (RT) (For details regarding the sampling procedure and terminology, see Fig. S1). The supernatant was used for quantification of nitrogen species and aqueous Fe(II) (Fe(II)_aq_).

### Analytical methods

N-species were quantified by an automatic method for colorimetric analysis using a continuous flow analyzer system (AA3, Seal Analytical, Norderstedt, Germany) (Anderson 1979). Nitrate was reduced to nitrite on an on-line copper cadmium reductor column, so that both nitrate and nitrite were quantified as azo dye photometrically at 520 nm, after reaction with sulfanilamide and *N*-(1-napthyl) ethylenediamine (Skeggs 1957). Ammonia was quantified as indophenol photometrically at 660 nm, after Berthelot reaction with dichloroisocyanuric acid and salicylate catalyzed by nitroprusside (Patton and Crouch 1977). Calibration curves were prepared with standards ranging from 0 to 7.5 mg/l of the corresponding N-compound. Therefore, samples from our experiments for nitrate and ammonia analyses were diluted in distilled water (aqua dest) before analysis. The dilution factor (11- or 21-fold) depended on the time point of sampling (corresponding to progress of nitrate reduction). Undiluted samples were used for nitrite quantification. Samples were stored anoxically at 4°C until analysis.

Fe_tot_, Fe(II)_tot_, and Fe(II)_aq_ were quantified by a colorimetric ferrozine-based microplate assay using a 96-well microplates and a microtiter plate reader (Multiskan GO Microplate Spectrophotometer, Thermo Fisher Scientific, Inc) (Stookey 1970, Schaedler et al. 2018). Fe(III) was reduced to Fe(II) using hydroxylammonium hydrochloride (HAHCl 10% w/v, 1 M HCl) by 30 min incubation at RT and therefore both total Fe and total Fe(II) (and total Fe(III) as the difference) were determined as ferrozine-Fe(II) complex photometrically at 562 nm, after reaction with the Ferrozine reagent [3-(2-pyridyl)-5,6-diphenyl-1,2,4-triazine-*p, p*′-disulfonic acid monosodium salt hydrate 1 g/l, 500 g/l ammonium acetate] for 5 min at RT, in the dark. The plate was amended with 80 µl of 1 M HCl or HAHCl and 20 µl sample then 100 µl ferrozine reagent were added per well. Each sample was measured in triplicates. A calibration curve was prepared with standards ranging from 0 to 1000 µM ammonium Fe(II) sulfate in SA: HCl (40 mM amidosulfonic acid, 1 M HCl).

The samples were diluted 21-fold in SA: HCl subsequently collected and stored at 4°C (Granger and Sigman 2009, Klueglein and Kappler 2013).

Fe(II)_tot_ and Fe(III)_tot_ in % were calculated using the results from Fe(II)_tot_ and Fe_tot_. Fe(III)_tot_ in mM was calculated by: $\frac{{Fe{{( {III} )}_{tot}}}}{{F{e_{tot}}}} \times 10\,\,\mathrm{ m}\mathrm{ M}$, where 10 mM is the initial Fe(II) concentration (for reasoning see supplemental information).

Cells were counted by flow cytometry using a fluorescence dye (BacLight Green bacterial stain, Invitrogen by Thermo Fischer Scientific, Inc.) and an Attune NxT connected to an autosampler (Thermo Fischer Scientific, Inc.). We observed that the Fe(III) mineral particles produced by Fe(II) oxidation in the cultures had a strong influence on the quality of cell counts. We established the following protocol to dissolve the iron precipitates. Pretest using cultures containing thiosulfate and nitrate (no minerals) showed that this protocol did not affect the staining efficiency of the applied fluorescent stain and the cell counting; data not shown. For consistency, the protocol was therefore applied to all samples, both with and without iron. Culture samples (200 µl) were mixed gently with 100 mM EDAS-Fe(II) (200 µl) and oxalic acid (600 µl: 28 g/l ${{\mathrm{C}}_{\mathrm{2}}}{{\mathrm{H}}_{\mathrm{2}}}{{\mathrm{O}}_{\mathrm{4}}}\,{\mathrm{ \times 2N}}\,{{\mathrm{H}}_{\mathrm{3}}}$, 15 g/l ${{\mathrm{(\mathrm{COOH})}}_{\mathrm{2}}}{\mathrm{ \times \, 2}}{{\mathrm{H}}_{\mathrm{2}}}{\mathrm{O}}$, pH 7.0). Subsequently, the samples were diluted 100-fold using oxalic acid and incubated for 30 min. The fluorescence dye (2 µl) was added and the samples were incubated in the dark for 15 min. The suspension was aliquoted to a 96-well microplate and analyzed as technical triplicates. One abiotic setup was sampled and served as negative control. The mean of the counts resulting from the corresponding three runs have been subtracted from the cell counts. Samples were acquired at a flow rate of 12 μl/min using 488 nm blue laser excitation sources and BL1 530/30 channel. Therefore, 25 µl of a 200 µl reservoir were used. The side scatter, forward scatter, and BL1 detector were set to 440 V, 350 V, and 380 V, respectively, and thresholds were set to 0.8 × 10^3^ fluorescence (BL1 530/30) intensity and 0.1 × 10^3^ side scatter intensity. Side scatter, forward scatter, and florescence were measured and reported as the height of the electronic pulse that comes off from the respective detector.

### D/L staining and fluorescence microscopy

LIVE/DEAD™ *Bac*Light™ Bacterial Viability Kit was used according to the manufacturer’s manual and the cell viability was evaluated by a fluorescence microscope (Leica CTR5500 Microscope, Leica Microsystems GmbH). The micrograph images were captured using a 20x objective lens and processed by Leica Application Suite X.

### Scanning electron microscopy

Samples for scanning electron microscopy (SEM) were collected from a *T. denitrificans* culture initially containing 3.5 mM nitrate and 10 mM Fe(II). Samples were withdrawn from the bottles anoxically with a syringe through a rubber stopper, while gently mixed by hand, inside of an anoxic glovebox, immediately after inoculation and after 5 days. Cells were fixed in 2.5% glutaraldehyde for at least 24 h at 4°C by adding 100 µl of a 25% glutaraldehyde solution to 900 µl of culture, under atmospheric conditions. After fixation, samples were centrifuged (1 min at 2348 × *g*), and 900 µl of supernatant was removed. The remaining sample was washed with 900 µl of ultrapure water, then centrifuged again (1 min at 2348 × *g*). This wash step was repeated twice. After the final wash, 900 µl of supernatant was removed, and the sample was diluted 1:1 with ultrapure water to achieve an optimal cell density for SEM.

For sample preparation, 50 µl of each sample was placed onto poly-l-lysine-coated cover glass slides (coated with 35 µl of 0.01% poly-l-lysine solution, PLANO, Wetzlar, item #18 026) and dried at 50°C for 2 h. The slides were arranged in a 24-well plate, covered with the plate lid, and left undisturbed for 30 min to allow cells to settle. Samples were then dehydrated through a graded ethanol series (25%, 50%, and 75% for 15 min each, followed by 3 × 100% for 30 min). Samples were transitioned to hexamethyldisilazane (HMDS) via a 1:1 ethanol/HMDS mixture for 30 min, followed by pure HMDS for another 30 min. Finally, 250 µl of fresh HMDS was added and allowed to evaporate overnight in a fume hood with the well plate lid slightly open.

Once dried, cover glass slides were mounted on aluminum stubs with carbon tape (PLANO, Wetzlar, item #G301 and G3347) and sputter-coated with ∼8 nm platinum using a BAL-TEC SCD 005 sputter coater. SEM imaging was conducted on a Zeiss Crossbeam 550 L FIB-SEM at an acceleration voltage of 2 kV and a working distance of 5.0 mm, utilizing the SESI detector for image capture.

### Kinetic model setup and parameter estimation

A kinetic model has been applied to support the hypothesis that *T. denitrificans* is a non-autotrophic nitrate-reducing Fe(II)-oxidizer, capable of performing the first step of denitrification, producing nitrite. This assumption is based on the experimental results of the present study and Beller et al. (2013).

Fe(II) oxidation stems from both enzymatic Fe(II) oxidation coupled to denitrification (equation 1) and abiotic oxidation due to nitrite as oxidizing agent (equation 2) (Tai and Dempsey 2009, Beller et al. 2013). The Fe(III) (oxyhydr)oxide mineral was assumed to be goethite in equation (2) based on previous work under similar experimental conditions albeit using different nitrate-reducing Fe(II)-oxidizers (Kappler et al. 2005) and based on the reaction mechanism suggested by Sørensen and Thorling (1991). The abiotic reduction of nitrite by aqueous Fe(II) was found to be faster in the presence of Fe(III) minerals because they promote the sorption of Fe(II), forming solid-bound Fe(II) which acts as a catalyst (Tai and Dempsey 2009, Van Cleemput and Baert 1983). Therefore the adsorption process of Fe(II)_aq_ on the Fe(III) (oxyhydr)oxide minerals (equation 3) effects the overall oxidation process.


(1) \begin{eqnarray*}
2{\mathrm{Fe}}({\mathrm{II}}) + {\mathrm{NO}}_3^ - + 2{{\mathrm{H}}^ + } \to 2{\mathrm{Fe}}({\mathrm{III}}) + {\mathrm{NO}}_2^ - + {{\mathrm{H}}_2}{\mathrm{O}}.
\end{eqnarray*}

(2) \begin{eqnarray*}
4{\mathrm{Fe}}({\mathrm{II}}) + 2{\mathrm{NO}}_2^ - + 5{{\mathrm{H}}_2}{\mathrm{O}} \to 4{\mathrm{FeO}}({\mathrm{OH}}) + {{\mathrm{N}}_2}{\mathrm{O}} + 6{{\mathrm{H}}^ + }.
\end{eqnarray*}

(3) \begin{eqnarray*}
{\mathrm{Fe}}({\mathrm{III}}){\mathrm{OH}} + {\mathrm{Fe}}({\mathrm{II}}){\mathrm{O}}{{\mathrm{H}}^ + } \to {\mathrm{Fe}}({\mathrm{III}}){\mathrm{OFe(II)OH + }}{{\mathrm{H}}^ + }.
\end{eqnarray*}

**Figure 2.**
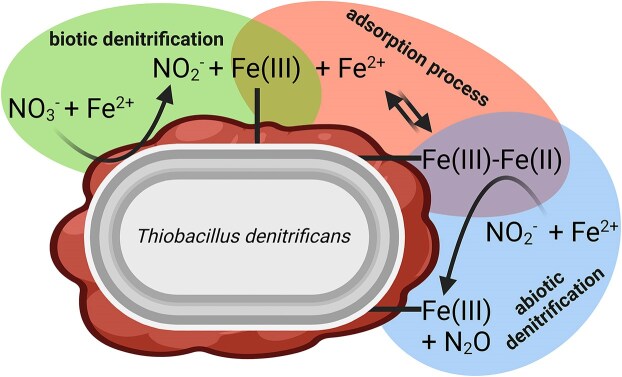
Conceptual model of enzymatic and abiotic denitrification catalyzed by *T. denitrificans*. Based on the experimental results of the presented study and Beller et al. (2013), *T. denitrificans* has been modeled as non-autotrophic nitrate-reducing Fe(II)-oxidizer performing the first step of denitrification (NO_3_^−^ →NO_2_^−^). Biotic denitrification with Fe^2+^ as electron donor produces nitrite and Fe(III) minerals (green background). Biotic denitrification with Fe^2+^ as electron donor produces nitrite and Fe(III) minerals (green background). Additional dissolved Fe^2+^ binds to Fe(III) mineral surfaces and both dissolved Fe^2+^ and sorbed Fe(II) reacts abiotically with nitrite producing N_2_O and Fe(III) minerals (red background). The schematic is based on the molecular formulas of equations (1–3), with stoichiometry omitted for simplicity.

The dynamic model described in the following scheme simplifies these underlying mechanisms (Fig. 2) and was applied to disentangle the biotic from the abiotic reaction. The simplifications are explained in detail in the supplemental information, but in brief it is important to note that we have modeled the change in total Fe(II)/(III). Modeling the change in aqueous Fe(II) is very complex due to unknown abiotic processes causing Fe(II) adsorption, precipitation, and dissolution. (for clarification on Fe phases please refer to Fig. S1).

The bacterial denitrification rates of Fe(II)_tot_ by NO_2_^−^ were simulated using dual-substrate Monod kinetics (equation 4). Since the progressive nitrate-reducing, Fe(II)-oxidizing activity of the bacteria can lead to encrustation of the cells by Fe(III) (oxyhydr)oxide minerals, an irreversible product inhibition by Fe(III) was added to the simulation of bacterial denitrification (Jamieson et al. 2018), leading to a rate equation of bacterial denitrification as follows:


(4) \begin{eqnarray*}
{r_{\textit{biotic}}} = {r_{{\mathrm{max}}}}\left( {\frac{{\left[ {{\mathrm{NO}}_3^ - } \right]}}{{\left[ {{\mathrm{NO}}_3^ - } \right] + {K_N}}}} \right)\left( {\frac{{\left[ {Fe{{\left( {II} \right)}_{tot}}} \right]}}{{\left[ {Fe{{\left( {II} \right)}_{tot}}} \right] + {K_{Fe}}}}} \right){f_{tox}},
\end{eqnarray*}

where *r*_max_ is the maximum denitrification rate, *K*_N_ and *K*_Fe_ is the half-saturation constant for NO_3_^−^ and Fe(II)_tot_, respectively, and ${f_{tox}}$ accounts for the toxicity term.

Following the assumption that abiotic denitrification occurs mainly on cell-bound Fe(III)/Fe(II) (oxyhydr)oxide minerals and thus additionally encrusts the cell (Jamieson et al. 2018), the toxicity term was made dependent on the concentration of total Fe(III). Four standard inhibition terms have been tested (see supplemental information) and a two-parameter logistic term performed best (Kalyuzhnyi et al. 1998, Poinapen and Ekama 2010, Belli et al. 2015, Robles et al. 2020):


(5) \begin{eqnarray*}
{f_{tox}} = \frac{1}{{1 + {{\left( {\frac{{\left[ {Fe{{\left( {III} \right)}_{tot}}} \right]}}{{I{C_{50}}}}} \right)}^{\frac{{{\mathrm{log}}\,\,\left( {99} \right)}}{{{\mathrm{log}}\,\,\left( {\frac{{{\mathrm{\,\,}}{K_{100}}}}{{I{C_{50}}}}} \right)}}}}}}
,
\end{eqnarray*}

where, K100 denote for the concentrations of Fe(III)_tot_ corresponding to a 100-fold decrease in bacterial denitrification rate and IC_50_ is an inhibitory constant representing the Fe(III)_tot_ concentration at which cell encrustation causes 50% (2-fold decrease) of inhibition. The exponent $\frac{{\rm log}\,\,({99})}{{\rm log}\,\,\left( \frac{K_{100}}{IC_{50}} \right)}$ determines the slope around the inflection point of IC_50_.

As previously noted, Fe(III) (equation 3) enhances the abiotic oxidation by nitrite (Tai and Dempsey 2009). Consequently, various forms of the rate function representing abiotic denitrification have been modeled to assess the relationship between Fe(III) concentration and the abiotic Fe(II) oxidation rate within the presented system (see supplemental information, Evaluation of the kinetic models). Equation (6), representing the kinetics for a heterogenous solution, was identified as the most suitable and was applied to model the data presented in this study. Although the use of an overall third order equation to model the underlying heterogeneous reaction was inspired by Tai and Dempsey (2009), it is noteworthy that here the concentration of Fe(II)_aq_ and the adsorbed fraction of Fe(II) were not used, but instead Fe(II)_tot_ and Fe(III)_tot_. (Tai and Dempsey 2009). It relies on the concentrations of Fe(II)_tot_, Fe(III)_tot_, and nitrite and it follows first-order kinetics with respect to these substrates:


(6) \begin{eqnarray*}
{r_{\textit{abtiotic}}} = {k_{{\mathrm{abtiotic}}}}\,\,\left[ {Fe{{\left( {II} \right)}_{tot}}} \right]\left[ {Fe{{\left( {III} \right)}_{tot}}} \right]{\left[ {{\mathrm{NO}}_2^ - } \right]^{}},
\end{eqnarray*}

with, *k*_abiotic_ representing the kinetic constant of the abiotic rate.

The coupled differential equations (7– 10) describe the rates of change of total ferrous and ferric iron, nitrate, and nitrite, respectively and were used to simulate the dynamics of these compounds during biotic and abiotic denitrification processes. The molecular ratios of the denitrification (equations 1 and 2) were implemented in the differential equations (7–10).


(7) \begin{eqnarray*}
\frac{{dFe{{\left( {II} \right)}_{tot}}}}{{dt}} = \,\, - \,\,2 \times {r_{\textit{biotic}}} - 4 \times {r_{\textit{abiotic}}}.
\end{eqnarray*}

(8) \begin{eqnarray*}
\frac{{dFe{{\left( {III} \right)}_{tot}}}}{{dt}} = \,\,2 \times {r_{\textit{biotic}}} + 4 \times {r_{\textit{abiotic}}}.
\end{eqnarray*}

(9) \begin{eqnarray*}
\frac{{dNO_3^ - }}{{dt}} = \,\, - \,\,{r_{\textit{biotic}}}.
\end{eqnarray*}

(10) \begin{eqnarray*}
\frac{{dNO_2^ - }}{{dt}} = \,\,\,\,{r_{\textit{biotic}}} - 2 \times {r_{\textit{abiotic}}}.
\end{eqnarray*}

The reaction parameters were estimated by fitting the model (equations 7–10) to the measured concentrations of Fe(II)_tot_, nitrate and nitrite. The simulation starts not at time point zero but after 1 day in order to omit the lag phase. The system was solved in MATLAB using the ODE solver ode15s (Shampine and Reichelt 1997).

For all the parameterizations the fitted parameter values were reported. The goodness of fit of the model is evaluated using normalized root-mean-square-error (NRMSE) (equation 11) (Liu et al. 2020).


(11) \begin{eqnarray*}
\textit{NRMSE} = {\mathrm{\,\,}}\frac{{\sqrt {\mathop \sum \nolimits_{i = 1}^n {{\left( {{y_{{\mathrm{model,}}i}} - {y_{{\mathrm{obs}},i}}} \right)}^2}/n} }}{{{y_{{\mathrm{obs,max}}}} - {y_{{\mathrm{obs}},{\mathrm{min}}}}}}.
\end{eqnarray*}

The model (7–10) was applied to estimate the parameters ${r_{max}};{K_N};{K_{Fe}};I{C_{50}};\rho {\mathrm{\,\,and\,\,}}{k_{{\mathrm{abtiotic}}}}$. The objective function is defined as equation (12),


(12) \begin{eqnarray*}
\mathop {\min }\limits_\theta \left( {f\left( \theta \right)} \right) = \mathop \sum \nolimits_{i = 1}^n {\left( {f\left( {\theta ,{x_i}} \right) - {y_{{\mathrm{obs}},i}}} \right)^2},
\end{eqnarray*}

where $\theta $ is the parameter vector; ${y_{{\mathrm{obs}},i}}$ the observations. The total number of observations for Fe(II)_tot_, nitrate and nitrite were 30. *lsqnonlin* algorithm in MATLAB was used for parameter optimization by minimizing equation (12). NRMSE was computed to evaluate the goodness of the fit (equation 11).

Ferric and ferrous iron solution speciation due to changes in pH up to Fe(II) oxidation were neglected as the system is considered to be buffered by the bicarbonate containing medium.

## Results

### Nitrate-reducing Fe(II) oxidation and growth of *T. denitrificans* cultures

**Figure 3.**
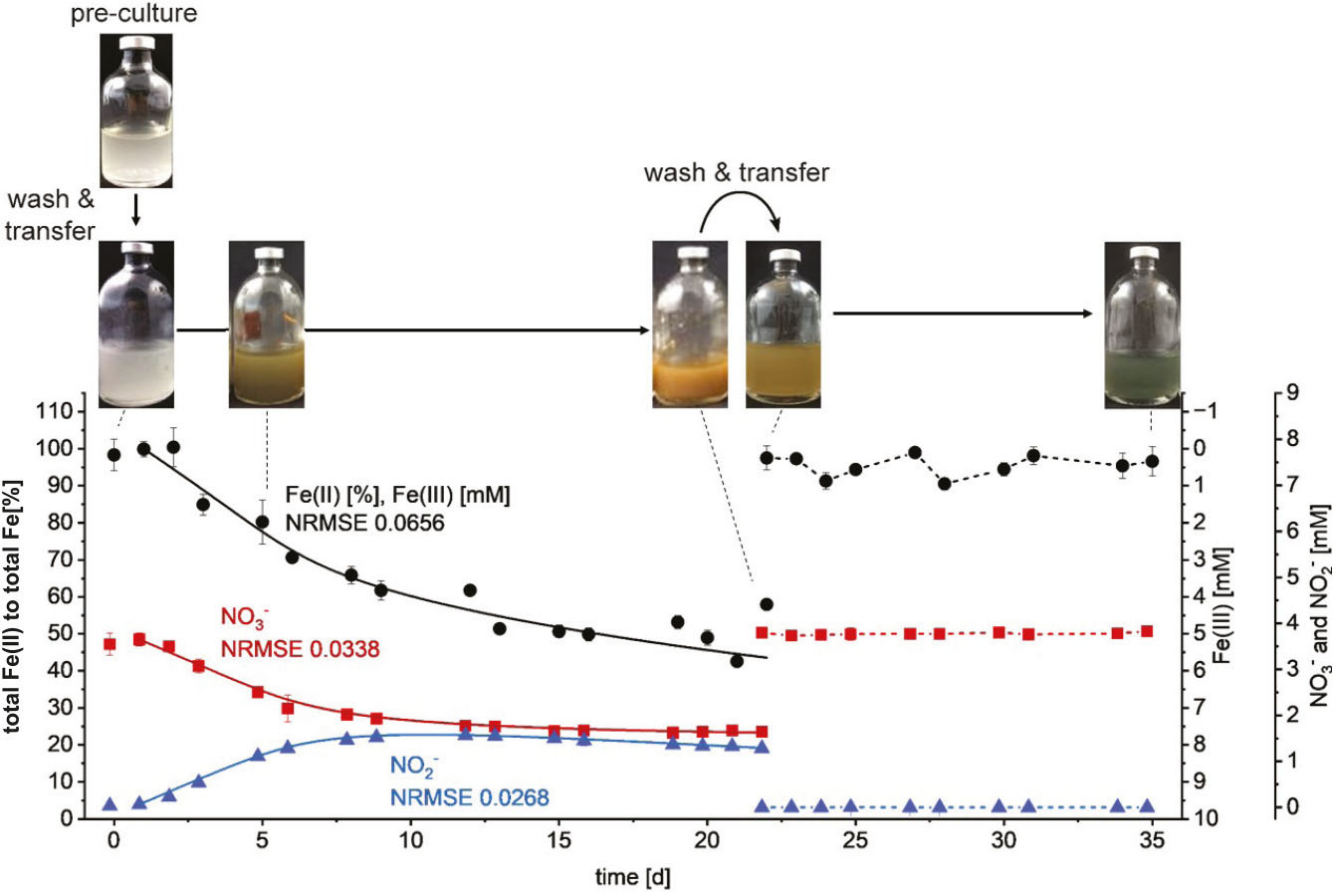
Total Fe(II) (black) and nitrate (red) consumption, along with nitrite production (blue), during nitrate-reducing Fe(II) oxidation by *T. denitrificans* in the first and second transfers with 3.5 mM nitrate and 10 mM Fe(II). Fe(II) oxidation is shown on the left *y*-axis as total Fe(II) as a percentage of the total Fe content in the culture, with the first right *y*-axis indicating the corresponding Fe(III) concentration in mM. Note that on day 22 the first transfer culture was used to inoculate fresh medium containing nitrate and Fe(II). Symbols represent the measured data and solid lines represent a kinetic model (equations 7–10). NRMSE: normalized root-mean-square-error. The selected photos of the serum bottles are representative of the replicates. Error bars indicate the standard deviation of biological replicates. Absence of error bars indicate that the error was smaller than the symbol size.

**Figure 4.**
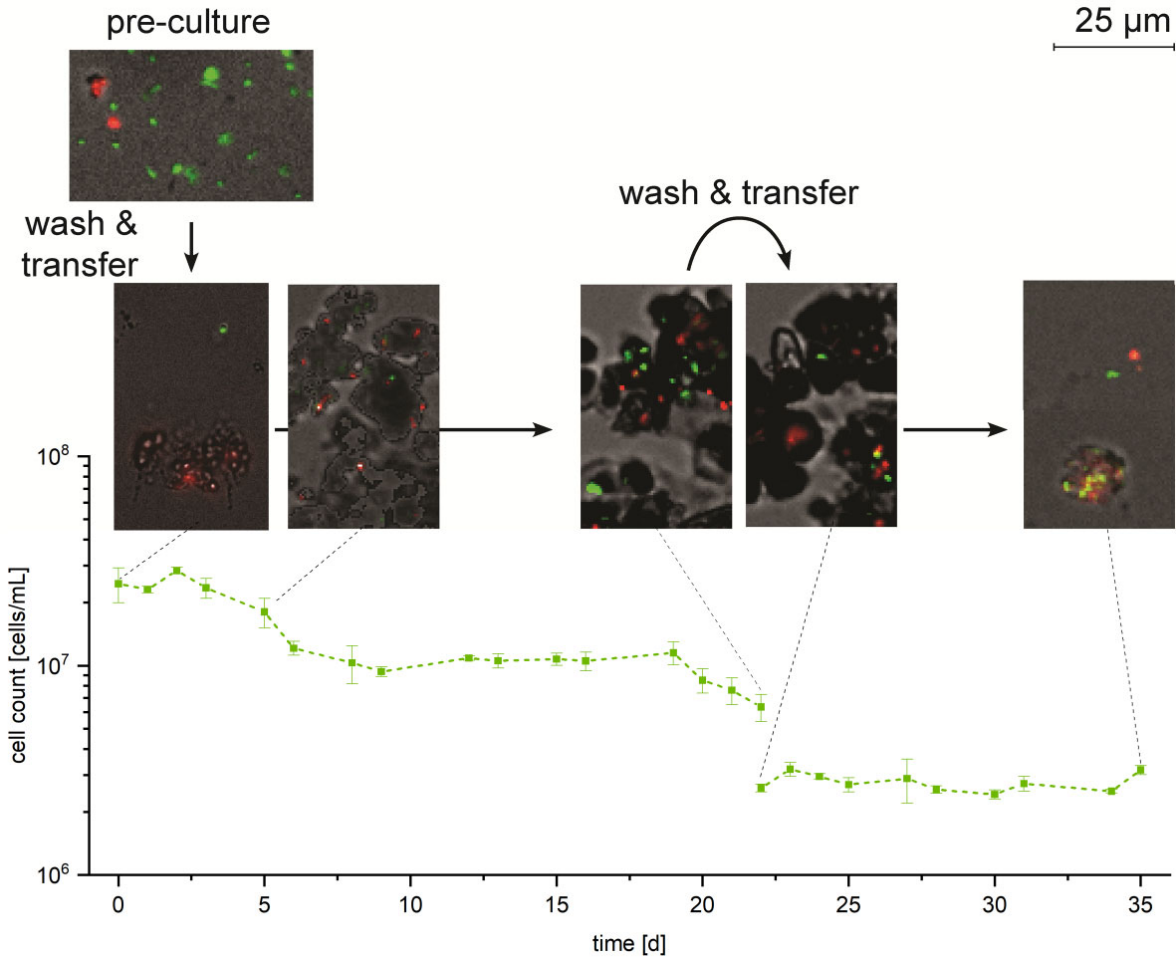
Cell counts of *T. denitrificans* in the first and second transfer cultures containing 3.5 mM nitrate and 10 mM Fe(II), with representative micrographs of the cultures at different time points. Note that on day 22 the first transfer, culture was used to inoculate fresh medium containing nitrate and Fe(II). Dead and defective cells are shown in red, live cells in green. While the cells for cell counting were treated with oxalic acid to dissolve iron, the samples for dead–live staining were untreated. Symbols represent the measured data and dashed lines connect them. Error bars indicate the standard deviation of biological replicates. Absence of error bars indicate that the error was smaller than the symbol size.

The growth experiment for *T. denitrificans* was conducted over two transfers. Initially, the culture (pregrown on thiosulfate and nitrate) was incubated in medium containing Fe(II) and nitrate for 22 days (transfer 1). The culture was then harvested and transferred to fresh medium containing again Fe(II) and nitrate (transfer 2) (Fig. 1). We found that in the first transfer *T. denitrificans* cultures with 10 mM Fe(II) and 3.5 mM nitrate, oxidized 42.02 ± 1.44% (4.20 ± 0.06 mM) total Fe(II), consumed 53.75 ± 3.23% (1.90 ± 0.06 mM) nitrate, and accumulated up to 1.56 ± 0.03 mM nitrite (44.17 ± 1.97% of the initial nitrate) within 22 days (Fig. 3). The cells did not grow, instead the cell number decreased to 25.80 ± 3.81% of the initial cell number (Fig. 4). The final cell number after 22 days was 38.81 ± 0.24% lower in setups containing both Fe(II) and nitrate than in cultures lacking either nitrate, Fe(II), or both (Fig. S2). The cell number in these cultures gradually decreased, averaging 67.04 ± 3.11% of the initial cell number after 22 days. Consequently, neither autotrophic growth utilizing Fe(II) as an electron donor nor heterotrophic growth due to either traces of vitamins stemming from the medium, stored organic carbon/reducing equivalents or low amounts of organic carbon present in the water used to prepare the medium was observed in any of the cultures (Rittenberg 1972). The positive growth control culture with thiosulfate as electron donor and nitrate as electron acceptor clearly demonstrated that the cells used as inoculum were not damaged by the harvest and washing procedure, as it grew similarly to the preculture (Fig. S3).

**Figure 5.**
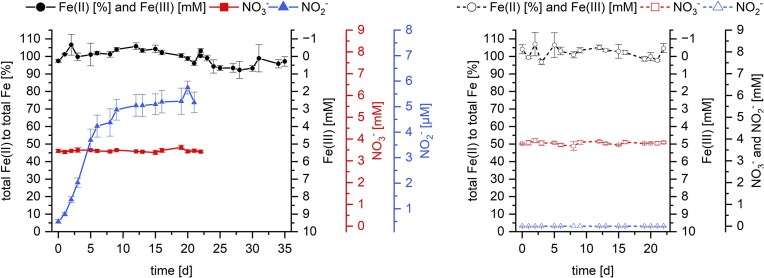
(A) Total Fe(II) oxidation and nitrate reduction in setups with *T. denitrificans* containing either 3.5 mM nitrate only (nitrate and nitrite data) or 10 mM Fe(II) only (iron data). Please note, the scale for nitrite is in µM. (B) Nitrate and Fe(II) over time in abiotic setups containing 3.5 mM nitrate plus 10 mM Fe(II) without cells. Fe is shown on the left *y*-axis as total Fe(II) as a percentage of the total Fe content in the culture, with the first right *y*-axis indicating the corresponding Fe(III) concentration in mM. Error bars indicate the standard deviation of triplicates. Absence of error bars indicate error was smaller than symbol size.

The *T. denitrificans* cultures containing nitrate and Fe(II) (first transfer from nitrate/thiosulfate) exhibited a lag phase of 1 day before they started oxidizing Fe(II) and reducing nitrate, with half of the nitrate and Fe(II) turnover occurring within ~5 days. In cultures containing Fe(II) and nitrate, nitrite accumulated to a maximum of 1.56 ± 0.03 mM after 12 days (Fig. 3). In contrast, in cultures with nitrate lacking Fe(II), nitrite accumulated only to a few µM (Fig. 5A). Nitrate reduction to ammonia was not observed in any of the cultures containing nitrate (see final ammonia concentrations in Table 1). No Fe(II) oxidation was observed in cultures containing Fe(II) but no nitrate (Fig. 5A). Neither abiotic nitrate reduction nor Fe(II) oxidation was observed in nitrate-/Fe(II)-containing controls without cells over the course of 22 days (Fig. 5B).

**Table 1.** Final abundance of nitrogen species, iron, and cells as well as the cell viability after 22 and 35 days of *T. denitrificans* incubations (see experimental setup in Fig. 1). First transfer of thiosulfate/nitrate pregrown bacteria to medium with 10 mM Fe(II) and 3.5 mM nitrate, and controls lacking one substrate or both, yielded in a cell number of initially ${\mathrm{4 \times 1}}{{\mathrm{0}}^{\mathrm{7}}}$ cells/ml. Second transfer of Fe(II)-oxidizing denitrifying bacteria to fresh medium again containing 10 mM Fe(II) and 3.5 mM nitrate, and control lacking nitrate or both, yielded in ${\mathrm{3 \times 1}}{{\mathrm{0}}^{\mathrm{6}}}$ cells/ml. The first incubation (first transfer) was conducted over 22 days, the second incubation (second transfer) was conducted for another 13 days (total 35 days). The values of iron, nitrate, ammonia, and cell number are given in percentages and represent the end-point concentrations relative to the initial concentration. While nitrite is given in percentage values as well, it is reported as the maximum nitrite accumulation (“peak”) relative to the initial nitrate concentration. Cell viability (cv) is categorized as + (culture appears healthy) or—(many cells showed defects), determined by dead–live staining. Bdl—below detection limit. [see sampling fraction of aqueous Fe(II) (Fe(II)_aq_) and total Fe(II) (Fe(II)_tot_) in Fig. S1].

Setup	Purpose	Transfer	Cell number (% of initial)	NH_4_^+^ (% of initial)	NO_3_^−^ (% of initial)	NO_2_^−^ peak (% of NO_3_^−^ initial)	Fe(II)_aq_ (% of initial)	Fe(II)_total_ (% of initial)	Cell viability
NO_3_^−^/Fe(II)/cells	Nitrate-reducing Fe(II) oxidation	1	25.80 ± 3.81	95.71 ± 1.62	46.25 ± 3.24	44.17 ± 1.97	15.94 ± 5.88	57.94 ± 1.44	–
		2	96.50 ± 1.95	107.90 ± 3.66	100.90 ± 2.57	bdl	71.88 ± 2.60	99.08 ± 4.06	–
NO_3_^−^/no Fe(II)/cells	Denitrification and growth due to stored carbon or vitamins	1	70.61 ± 4.23	97.15 ± 0.58	99.01 ± 1.35	0.15 ± 7.86			+
No NO_3_^−^/Fe(II)/cells	Nitrate-independent Fe(II) oxidation	1	65.59 ± 4.03				83.57 ± 4.66	103.22 ± 1.22	+
		2	109.36 ± 4.00				75.37 ± 0.37	96.81 ± 2.72	–
No NO_3_^−^/no Fe(II)/cells	Growth due to stored carbon or vitamins	1	64.92 ± 12.53						+
		2	53.78 ± 10.74						–
NO_3_^−^/Fe(II)	Role of the bacteria in the observed process	1		102.41 ± 2.09	101.50 ± 1.44	bdl	55.42 ± 15.95	100.61 ± 2.45	
NO_3_^−^/thiosulfate	Positive control to prove the use of an intact culture	1	5150						+
		2	106.41						–

After the second transfer (using inoculum harvested after 22 days from the Fe(II)/nitrate setup) the cultures did not show any denitrifying or Fe(II)-oxidizing activities (Fig. 3). Furthermore, the culture did not even grow anymore in the bottles intended to serve as positive growth control (transferred after 22 days from Fe(II)/nitrate to thiosulfate/nitrate) (Fig. S3).

To better understand the processes occurring in the cultures, in addition to total Fe(II) we also analyzed the fraction of dissolved Fe(II), i.e. aqueous Fe^2+^. A decrease in aqueous Fe^2+^ was observed not only in the Fe(II)-oxidizing denitrifying culture (decrease of 84.06 ± 5.89%; 6.46 ± 0.38 mM) at the first transfer but also in the abiotic control (decrease of 44.58 ± 15.96%; 3.39 ± 1.23 mM) and in cultures of the second transfer (decrease of 28.12 ± 2.60%; 2.11 ± 0.05 mM) (Fig. S4; final concentration Table 1). Biotic and abiotic processes occurring in the cultures are also evident from the changes in the cultures’ colors (Fig. 3). During the first transfer, cultures containing nitrate and Fe(II) showed first white particles (probably precipitates of Fe^2+^ with phosphate and carbonate yielding vivianite- and siderite-related minerals; ThomasArrigo et al. 2017), which turned green and then orange as the experiment progressed indicating Fe(II) oxidation. In the second transfer, where no further Fe(II) oxidation occurred, the color changed from initially orange (stemming from the Fe(III) (oxyhydr)oxide minerals transferred together with the inoculum) first to brown and finally to dark blue–gray indicating abiotic iron mineral transformation, probably caused by the dissolved Fe^2+^ and sorbed Fe(II) (Hansel et al. 2003).

### Mineral precipitation on the cell surface revealed by SEM

**Figure 6.**
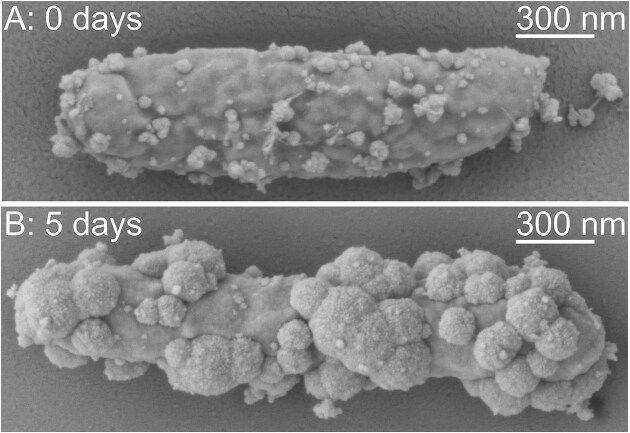
Scanning electron micrographs of a single *T. denitrificans* cell, the culture was pregrown on 20 mM thiosulfate and 20 mM nitrate, washed and transferred to medium containing 10 mM Fe(II) and 3.5 mM nitrate. (A) Immediately after inoculation and (B) after 5 days of incubation, at which 2.42 mM of Fe(III) was formed [24.19% oxidation of 10 mM Fe(II)].

To investigate cell association with minerals or even potential cell encrustation, *T. denitrificans* was precultured in medium with 20 mM thiosulfate and 20 mM nitrate, then washed and transferred to a medium containing 10 mM Fe(II) and 3.5 mM nitrate. The cells corresponding to the “immediately after inoculation” sampling time point had a few, small mineral precipitates on their surfaces (Fig. 6A). This artifact was expected given that sample preparation was performed under atmospheric conditions, allowing small amounts of Fe(II) to be oxidized by atmospheric oxygen to form Fe(III) minerals, which adhere to the cell surfaces. After 5 days, the culture contained 2.42 mM Fe(III), 2.11 mM nitrate, and 1.34 mM nitrite, with mineral precipitates observed on cell surfaces, leading to partial cell encrustation (Fig. 6B).

### Comparison of total Fe(II) oxidation rates of *T. denitrificans* under autotrophic conditions versus mixotrophic NRFeOx under mixotrophic conditions

During the first transfer of *T. denitrificans* from thiosulfate/nitrate on Fe(II)/nitrate, the overall Fe(II) oxidation rate was found to be about four times slower than the Fe(II) oxidation rates derived from mixotrophic NRFeOx cultures (*Acidovorax* strains BoFeN1, *Paracoccus denitrificans* strain Pd1222, and *Pseudogulbenkiania* strain 2002) reviewed by Jamieson et al. (2018).

Incubation of these NRFeOx under mixotrophic conditions (Fe(II) plus organic electron donor and nitrate as electron acceptor), resulted in an overall Fe(II) oxidation rate of $\sim 2\,\,\frac{{mM}}{d}$ on average [including partial enzymatic Fe(II) oxidation by the cells and the abiotic Fe(II) oxidation by nitrite formed during heterotrophic denitrification], whereas the Fe(II) oxidation rate obtained for *T. denitrificans* in our study has been $\sim 0.5\,\,\frac{{mM}}{d}$. The initial cell number of our experiment and the cell numbers in the experiments reviewed by Jamieson et al. (2018) are comparable, although the cell numbers in their experiments increased over time under mixotrophic conditions (Carlson et al. 2013, Klueglein and Kappler 2013, Jamieson et al. 2018). In contrast, the mixotrophic culture experiment using *Acidovorax* sp. BoFeN1 from Muehe et al. (2009 showed with $1 \times {10^{ - 11}}\frac{{mM}}{{\textit{cell} \times d}}$ a similar Fe(II) oxidation rate per cell as the *T. denitrificans* culture in the herein presented study were the rate has been $2 \times {10^{ - 11}}\frac{{mM}}{{\textit{cell} \times d}}$.

### Kinetic model for nitrate-reducing Fe(II) oxidation by *T. denitrificans* and abiotic denitrification

A kinetic model was employed to analyze the stoichiometry of the redox reactions during the first transfer of *T. denitrificans* from thiosulfate/nitrate to Fe(II)/nitrate and to estimate the share of bacterial (enzymatic) and abiotic Fe(II) oxidation. The dynamic model was calibrated against the measured nitrate, nitrite, and total Fe(II) concentrations of the Fe(II)-oxidizing, denitrifying culture. The parameters $\,\,({r_{max}};{K_N};{K_{Fe}};I{C_{50}};\rho {\mathrm{\,\,and\,\,}}{k_{{\mathrm{abtiotic}}}}$) of the model (equations 7–10) are listed in Table 2.

**Table 2.** Parameters resulting from the kinetic model describing nitrate-reducing Fe(II) oxidation by *T. denitrificans* (equations 4– 6). Note, this is the result of a mathematical model, and not half-saturation constants determined using an enzyme assay. The presented half-saturation constants have a mathematical, however, no conceptual physical meaning.

Fitted parameter	Constant
Maximum denitrification rate (r_max_)	$0.28\,\,\frac{{mM}}{d}$
Half-saturation constant for nitrate (K_N_)	$10\,\, \times {10^{ - 160}}\,\,mM$
Half-saturation constant for Fe(II) (K_Fe_)	$2\,\, \times {10^{ - 31}}\,\,mM$
Abiotic kinetic rate constant (k_abiotic_)	$0.64\,\, \times {10^{ - 3}}\,\,\frac{1}{{m{M^2} \times d}}$
Inhibitory constant, 100-fold (K_100_)	$6.88\,\,mM$
Inhibitory constant, 50% (IC50)	$3.19\,\,mM$

The half-saturation constants ${K_N}\,\,$and ${K_{Fe}}$ of the Monod terms, predicting biotic denitrification, approach zero (equation 4). In the frame of the experiment, the concentrations of nitrate and iron do not approach zero since they are not fully consumed; consequently, the Monod terms approach one, implying that the substrate concentration remains high and thus the denitrification rate is near-maximum. Noteworthy, different starting points and boundaries for the half saturation constants where tested, and either the estimated half saturation constants approached zero or were in the range of 10^3^ while the maximum rate increased. For both outcomes, the term “${r_{{\mathrm{max}}}}( {\frac{{[ {{\mathrm{NO}}_3^ - } ]}}{{[ {{\mathrm{NO}}_3^ - } ] + {K_N}}}} )( {\frac{{[ {Fe{{( {II} )}_{tot}}} ]}}{{[ {Fe{{( {II} )}_{tot}}} ] + {K_{Fe}}}}} )$” of equation (4) remained the same. Therefore, the kinetics of nitrate-reducing Fe(II) oxidation rather shows linear behavior, as opposed to following Monod kinetics. While the Monod terms approach one, the rate is primarily dictated by the toxicity term. This term has a strong influence on the rate, especially outside the initial near-linear phase. This dominant behavior of the toxicity function has been observed for all tested inhibition terms (see supplemental information).

The simulated concentrations of nitrate, nitrite, and Fe(II) are plotted as lines in Fig. 3. The NRMSE of nitrate, nitrite, and total Fe(II) data were calculated to be 0.038, 0.034, and 0.069, respectively, demonstrating a good fit. Therefore, the model supports the prevailing hypothesis that two Fe(II) ions are oxidized coupled to the reduction of one nitrate molecule to nitrite through bacterial denitrification (equation 1), and subsequently, two nitrite molecules react with four Fe(II) through abiotic denitrification (equation 2). Consequently, the model corroborates the notion that Fe(II) is not further utilized as an electron donor for CO_2_ fixation.

**Figure 7.**
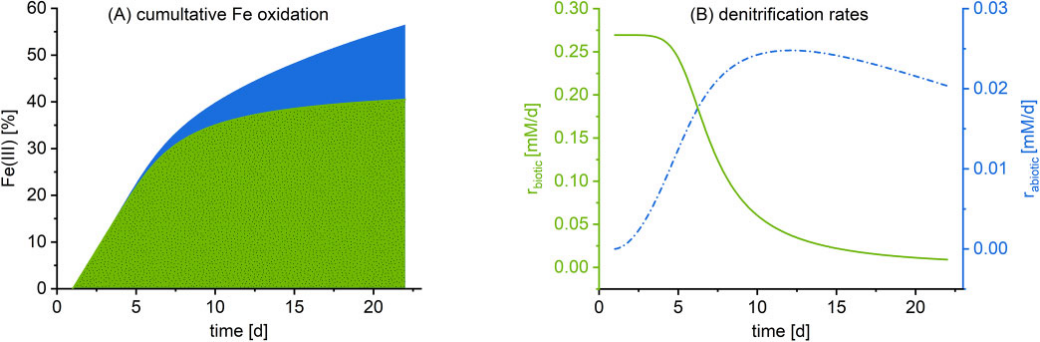
(A) Cumulative Fe(II) oxidation and denitrification rates simulated and based on the model of equations (7–10). The overall Fe(II) oxidation is based on bacterial Fe(II) oxidation (green) and abiotic Fe(II) oxidation by nitrite (blue). (B) Corresponding biotic (green) and abiotic (blue/dashed line) denitrification rates. The biotic reaction is characterized by a Monod function and a two-parameter logistic toxicity term (equation 7), while the abiotic denitrification follows a first-order kinetic with respect to Fe(II), Fe(III), and nitrite (equation 9).

Furthermore, the model was applied to estimate the reaction rates in order to disentangle biotic and abiotic Fe(II) oxidation. The bacterial denitrification starts at its maximum, where ${r_{\textit{biotic}}}$ is $0.27\,\,\frac{{mM}}{d}$ and the abiotic reaction rate reaches a maximum after 10.7 days, with ${r_{\textit{abiotic}}}$ of $0.025\,\,\frac{{mM}}{d}$. This equals to $0.54\,\,\frac{{mM}}{d}$ biotic Fe(II) oxidation rate ($2 \times {r_{\textit{biotic}}}$) and $0.10\,\,\frac{{mM}}{d}$ abiotic Fe(II) oxidation rate ($4 \times {r_{\textit{abiotic}}}$), respectively. The maximum Fe(II) oxidation rate of the abiotic reaction is 5.4 times slower than the maximum biotic Fe(II) oxidation rate (Fig. 7).

Based on the applied model, 70% of Fe(II) oxidation are due to enzymatic oxidation and 30% are attributed to abiotic reactions with nitrite (Fig. 7).

In the following (Table 3), the kinetics of different kinetic models were applied to determine abiotic Fe(II) oxidation rates using the setting of two time points of interest of the *T. denitrificans* Fe(II)/nitrate system studied. The first time point of interest has been at the maximum abiotic Fe(II) oxidation rate determined by the use of the herein presented model (equations 7–10) at 10.80 days, with a rate of $0.1\frac{{mM}}{d}$. Approaching the end of the incubation, the bacteria are inactive and only abiotic oxidation is observed, this has been the second time point of interest. The abiotic Fe(II) oxidation rate at 22 days, end of the experiment, was $0.08\,\,\frac{{mM}}{d}$. The maximum Fe(II) oxidation rate and the rate at the end of this experiment were found to be about eight times slower to 2.4 times faster than the Fe(II) oxidation rates derived from the kinetics based on abiotic Fe(II)/nitrite experiments using bicarbonate-buffered medium (BBM) without bacteria ($0.01 - 0.24\,\,\frac{{mM}}{d}$, Table 3) (Jamieson et al. 2018). The rates derived from the kinetics based on experiments without bicarbonate buffer of homogeneous systems [Fe(II)/nitrite] were up to 47 times faster ($4.7\,\,\frac{{mM}}{d}$, Table 3) (Jones et al. 2015) and of a heterogeneous system [Fe(III)/Fe(II)/nitrite] up to 234 times faster ($23.36\,\,\frac{{mM}}{d}$, Table 3) (Tai and Dempsey 2009).

**Table 3.** Comparison of different kinetic models for abiotic Fe(II) oxidation by nitrite and the corresponding rates. The rates represent abiotic Fe(II) oxidation at 10.8 and 22 days in *T. denitrificans* cultures grown in BBM, initially containing 10 mM Fe(II) and 3.5 mM nitrate. The cultures were inoculated using a preculture grown on thiosulfate and nitrate. Substrate concentrations used to calculate the specific rates for the listed equations were derived from the applied model (equations 7–10) for consistency. Since the model does not account for changes in aqueous Fe(II), the measured aqueous Fe(II) concentration from day 11 was used. Fe(II)_tot_—total iron (solid and aqueous); Fe(II)_aq_—aqueous iron; BBM—bicarbonate-buffered medium; PBM—PIPES-buffered medium. [See sampling fraction of aqueous Fe(II) (Fe(II)_aq_) and total Fe(II) (Fe(II)_tot_) in Fig. S1].

Experimental setup	Equation	*k*	*t* [d]	${r_{Fe}}$ [$\frac{{mM}}{d}$]	Reference
*T. denitrificans*: BBM/Fe(II)/nitrate	${r_{Fe}} = k\,\,[ {Fe{{( {II} )}_{tot}}} ][ {Fe( {III} )} ][ {{\mathrm{NO}}_2^ - } ]$	$2.56 \times {10^{ - 3}}\,\,\frac{1}{{m{M^2} \times d}}$	10.80	0.1	This study
			22.00	0.08	
Culture HP: BBM/Fe(II)/nitrate	${r_{Fe}} = k\,\,[ {Fe{{( {II} )}_{tot}}} ][ {{\mathrm{NO}}_2^ - } ]$	$0.05\frac{1}{{mM \times d}}$	10.80	0.53	H. Grimm unpublished data culture HP (Gimm et al. 2024)
			22.00	0.31	
Abiotic: filtered BBM/Fe(II)/nitrite	${r_{Fe}} = k\,\,[ {Fe{{( {II} )}_{aq}}} ][ {{\mathrm{NO}}_2^ - } ]$	$0.69 \times {10^{ - 2}}\,\,\frac{1}{{mM \times d}}$	10.80	0.02	Dopffel et al. (2021)
			22.00	0.01	
Abiotic: citrate-BBM/Fe(II)/nitrite	${r_{Fe}} = k\,\,[ {Fe{{( {II} )}_{aq}}} ][ {{\mathrm{NO}}_2^ - } ]$	$8.50 \times {10^{ - 2}}\,\,\frac{1}{{mM \times d}}$	10.80	0.23	Kopf et al. (2013), Jamieson et al. (2018)
			22.00	0.14	
Abiotic: BBM/Fe(II)/nitrite	${r_{Fe}} = k\,\,[ {Fe{{( {II} )}_{aq}}} ][ {{\mathrm{NO}}_2^ - } ]$	$2.10 \times {10^{ - 2}}\,\,\frac{1}{{mM \times d}}$	10.80	0.05	Klueglein and Kappler (2013), Jamieson et al. (2018)
			22.00	0.03	
Abiotic: PIPES/Fe(II)/nitrite	${r_{Fe}} = k\,\,[ {Fe{{( {II} )}_{aq}}} ][ {{\mathrm{NO}}_2^ - } ]$	$16.07 \times {10^{ - 2}}\,\,\frac{1}{{mM \times d}}$	10.80	0.43	Jones et al. (2015), Jamieson et al. (2018)
			22.00	0.26	
Abiotic: PIPES/Fe(II)/nitrite	${r_{Fe}} = k\,\,[ {Fe{{( {II} )}_{tot}}} ]$	$0.74\,\,\frac{1}{d}$	10.80	4.7	Jones et al. (2015)
			22.00	3.56	
Abiotic: PIPES/Fe(II)/Fe(III)/nitrite	${r_{Fe}} = k\,\,[ {Fe{{( {II} )}_{aq}}} ][ {{\mathrm{NO}}_2^ - } ]$	$8.74\,\,\frac{1}{{mM \times d}}$	10.80	23.36	Tai and Dempsey (2009)
			22.00	13.98	
Abiotic: PBM/Fe(II)/nitrite	${r_{Fe}} = k\,\,[ {Fe{{( {II} )}_{tot}}} ][ {{\mathrm{NO}}_2^ - } ]$	$22.80 \times {10^{ - 2}}\,\,\frac{1}{{mM \times d}}$	10.80	2.41	Liu et al. (2019)
			22.00	1.42	
Abiotic: MOPS/Fe(II)/nitrite	${r_{Fe}} = \frac{{[Fe{{( {II} )}_{aq}}] - ( {{{[Fe{{( {II} )}_{aq}}]}^{k \times t}}} )}}{t}$ with $t\,\, = 1\,\,d$	$0.98\,\,\frac{1}{d}$	10.80	1.01	Chen et al. (2020)
			22.00	0.40	
**10.8 days: 6.45 mM Fe(II)_tot_; 1.63 mM Fe(II)_aq_; 3.55 mM Fe(III)_tot_; 1.64 mM NO_2_^−^**					
**22 days:4.8 mM Fe(II)_tot_; 1.23 mM Fe(II)_aq_; 5.2 mM Fe(III)_tot_; 1.3 mM NO_2_^−^**					

## Discussion

### *Thiobacillus denitrificans*: a non-autotrophic NRFeOx and its inability to grow with Fe(II) as the sole electron donor

While *T. denitrificans* harvested from a culture growing with thiosulfate and nitrate demonstrated the capability for nitrate-reducing Fe(II) oxidation, it was incapable of autotrophic growth when Fe(II) serves as the sole electron donor and CO_2_ as the sole carbon source. Therefore, *T. denitrificans* is not an autotroph with respect to nitrate-reducing Fe(II) oxidation, or in other words, it does not function as a chemolithoautotrophic NRFeOx alone. These findings stand in contrast to Straub et al. (1996) reporting that *T. denitrificans* growths on nitrate and Fe(II), autotrophically. However, sufficient supportive data for autotrophic growth were not demonstrated by Straub et al. (1996).

Further, the observations of our study agree with Beller et al. (2013) who demonstrated a strong correlation of Fe(II) oxidation and nitrate reduction by a whole-cell suspension assay and performed a cultivation experiment over a period of 20 days to evaluate autotrophic growth. Based on the ratio of reduced nitrate to oxidized Fe(II), Beller et al. (2013) suggested incomplete denitrification stopping at nitrite, although they were unable to quantify the nitrite concentration because it was under the detection limit of their method. As indicator for growth, Beller et al. (2013) monitored the protein concentration and no growth was found. In the present study, higher substrate concentrations enabled the detection of nitrite, confirming that *T. denitrificans* reduced nitrate only to nitrite with Fe(II) as electron donor. Although it is possible that a small amount of nitrite could be reduced to N₂ later during the experiment, this outcome would alter the electron balance applied in the model presented here (Fig. 3), which uses stoichiometry for biological reduction to nitrite followed by abiotic reduction to N₂O (equations 1 and 2). Given the model’s strong fit, we conclude that nitrite is abiotically converted to N₂O, which was not quantified in this study. Based on multiple control setups and direct cell counts our data clearly demonstrates that *T. denitrificans* cannot grow under autotrophic nitrate-reducing Fe(II)-oxidizing conditions.

### Implications from the modeling results

#### Lesson learned about the diversity of kinetic models reflecting chemodenitrification

Various methods have been used to differentiate abiotic from biotic Fe(II) oxidation during nitrate-reducing Fe(II) oxidation, such as temperature-based experiments (Dopffel et al. 2021), comparing different mixotrophic strains (Jamieson et al. 2018), N₂O monitoring (Jones et al. 2015), and abiotic batch experiments (Tai and Dempsey 2009, Chen et al. 2020). The present study used a novel approach by extending experiments to a point where cell activity ceased, allowing for a clearer distinction between biotic and abiotic processes. As the kinetic model of this study led to comparable Fe(II) oxidation rates than rates derived from abiotic systems using BBM, the model succeeded to distinguish the abiotic from the biotic Fe(II) oxidation. Oxidation rates derived from BBM systems are slower than oxidation rates of systems without bicarbonate (Table 3) (Jamieson et al. 2018, Dopffel et al. 2021). Oxidation rates of heterogeneous reactions are faster than derived from homogeneous reactions (Table 3) (Tai and Dempsey 2009, Jones et al. 2015). This study and the study of Jamieson et al. (2018) indicate that the kinetics observed from abiotic homogeneous experiments in BBM are transferable for modeling nitrate-dependent Fe(II) oxidation in BBM systems. The slower Fe(II) oxidation rates derived from BBM systems may be a consequence of initially not having all iron(II) in solution but having a mix of solid and dissolved Fe(II) in the system due to Fe(II) mineral precipitation as siderite and vivianite as a consequence of the presence of bicarbonate (as buffer) and phosphate (as nutrient) in the medium. Further, the rates derived from a citrate BBM system studied by Kopf et al. (2013) are up to 12 times faster than the rates from setups without an organic acid (Table 3) (Dopffel et al. 2021, Klueglein and Kappler 2013). By studying the effect of citrate, Kopf et al. (2013) aimed to mimic the biological environment. However, it has been shown that bacteria secrete metabolites which, in contrast, lower abiotic Fe(II) oxidation (Baker et al. 2023). As the rates derived from this study’s kinetic model approximate the average of the rates corresponding to abiotic BBM systems (with and without citrate), it indicates that complexation to organic matter plays a minor role in the abiotic Fe(II) oxidation in the presented system.

#### Lesson learned about the kinetics of nitrate-reducing Fe(II) oxidation and why Monod kinetics do not apply

The initial kinetics of nitrate-reducing Fe(II) oxidation observed in our experiments are linear and do not following Monod kinetics (equation 4), as the Monod terms approach 1 while the half-saturation constants approach zero. Similar to Jamieson et al. (2018), nitrate and Fe(II) concentrations had minimal influence on biotic denitrification rates, suggesting bacterial activity is primarily controlled by inhibition (Jamieson et al. 2018). In general, the Monod term approaches the maximum rate while the bacteria, seen as whole cell biocatalyst, are saturated by their substrates. When an experiment is performed where the substrate concentrations are not decreased low enough to observe a status where the bacteria are not saturated, it is not possible to predict half-saturation constants. As the half-saturation constants ${K_N}\,\,$and ${K_{Fe}}$, obtained by the herein applied model, approach zero while the substrate concentrations at the end of the experiment are 5 mM Fe(II) and 1.6 mM nitrite, these constants do not have a physical but only a mathematical meaning.

#### Lesson learned about a sudden inhibition of nitrate-reducing Fe(II) oxidation

A noncompetitive inhibition term based on total Fe(III) concentration (Table S1) best describes nitrate-reducing Fe(II) oxidation by *T. denitrificans*. The model suggests a sudden event with a significant limiting effect on the denitrification rate, which could be due to cell encrustation (Fig. 7B). However, since Fe(III) mineral encrustation is a gradual process, it is unlikely to cause such an abrupt inhibition. While the inhibition term based on Fe(III) aligns well with the data, this is not definitive proof that Fe(III) encrustation is responsible for the inhibition. The likelihood of encrustation as the cause is discussed further in the next section, alongside alternative scenarios involving sudden inhibition events.

#### Lesson learned about the Fe(II)/nitrate ratio and implications for electron usage in denitrification

As the applied model considers a Fe(II)_oxidized_/nitrate_reduced_ (Fe/N) ratio of 2 and fits well to the measured data, we can conclude that *T. denitrificans* does not use Fe(II) as electron donor for CO_2_ fixation. Our data rather support the observation that two Fe(II) ions are oxidized while one nitrate molecule is reduced to nitrite during bacterial denitrification. There is a strong correlation between Fe(II) oxidation and nitrate reduction, reflected in the consistent 2:1 ratio. However, we cannot definitively state whether the electrons from Fe(II) reduce nitrate directly or if they drive a biochemical reaction that causes further downstream of the metabolism of *T. denitrificans* the reduction of nitrate by another reducing agent such as NADH stemming from the tricarboxylic acid cycle. At this state, the observed 2:1 ratio is an overall ratio.

In summary, the three processes applied to the model were sufficient to provide a good fit and therefore probably capture the dominant processes in the *T. denitrificans*, Fe(II)/nitrate system presented here. These processes were (i) biological denitrification (NO_3_^−^→NO_2_^−^), (ii) the increasing inhibition of the first process, in the course of Fe(II) oxidation, and (iii) abiotic denitrification (NO_2_^−^→N_2_O), which is autoinduced by Fe(III) mineral production.

### Challenging the autotrophic paradigm in nitrate-reducing Fe(II) oxidation

In the early period after the discovery of nitrate-reducing Fe(II) oxidation (NRFeOx), the assumption was that this process is intrinsically linked to autotrophy. However, nowadays we know that this is not always the case. This section will use the data obtained in the presented study to discuss why nitrate-reducing Fe(II) oxidation has historically been associated with autotrophic growth and explore the knowledge gaps that persist in this field (Straub et al. 1996, Bryce et al. 2018).

**Figure 8.**
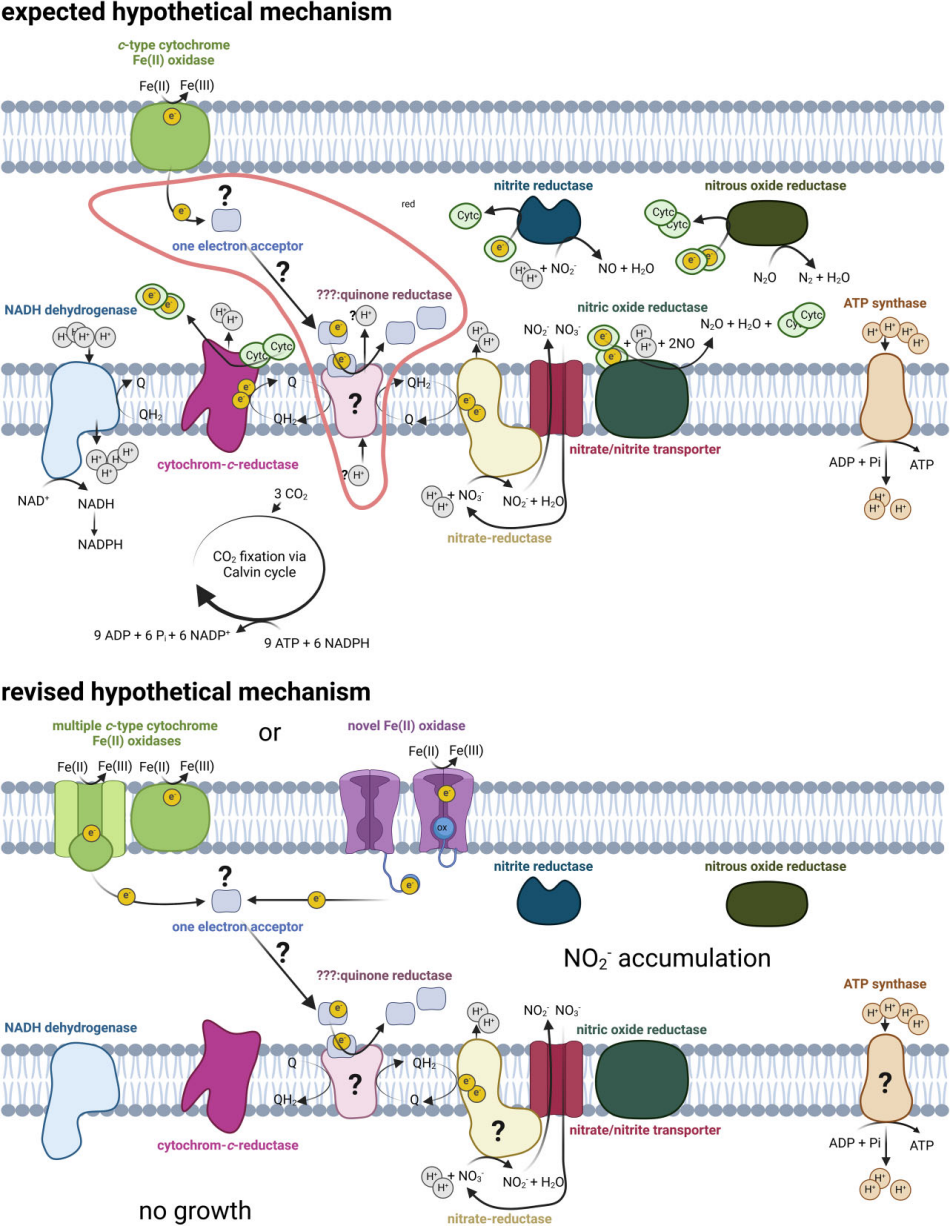
Comparison of a model autotrophic nitrate-reducing Fe(II)-oxidizing metabolic pathway (top) with a revised non-autotrophic version (bottom), based on the findings of Beller et al. (2013) and the present study. The electron transport pathway highlighted in red indicates the significant knowledge gap regarding how Fe(II)-derived electrons are transferred to the quinone pool. The proton (H^+^) stoichiometry is based on Simon et al. (2008) and Steigmiller et al. (2008). Cytochrome-*c*-reductase: cytochrome *bc*_1_ complex.

The prevailing hypothesis is that Fe(II) is oxidized by *c*-type cytochrome outer membrane proteins, such as MtoA or Cyc2, which are commonly found in the geno- and phenotypes of microaerophilic and nitrate-reducing Fe(II)-oxidizing bacteria (Garber et al. 2020, McAllister et al. 2020, Huang et al. 2021a) (Fig. 8). However, *T. denitrificans* lacks homologs of these classical iron oxidases. Beller et al. (2013) hypothesized that another *c*-type cytochrome might act as the initial electron acceptor. Given that Fe(II) has been shown to reduce the quinone pool (Tian et al. 2024), a logical assumption is that Fe(II)-derived electrons accepted by redox active proteins are transferred to the quinone pool. However, the mechanism by which this occurs remains poorly understood and is a significant knowledge gap in the literature (Becker et al. 2021).

Quinones play a key role in metabolic flexibility and rapid adaptation to environmental changes. In the downhill electron transport pathway, the quinone pool is used to the generate a proton motive force (pmf), which is subsequently utilized to regenerate adenosine triphosphate (ATP). In the uphill pathway (reverse electron transport), electrons derived from the quinone pool, along with the pmf, are used to regenerate nicotinamide adenine dinucleotide phosphate, reduced form (NADPH), a critical reducing agent for anabolic processes, including CO_2_ fixation during autotrophy. Based on this model, it appears that Fe(II)-derived electrons could support autotrophic CO_2_ fixation.

However, this idealized view of autotrophic NRFeOx must be revised in light of the findings from both Beller et al. (2013) and the herein presented study. Beller et al. (2013)’s knockout studies aiming at identifying the specific Fe(II) oxidase in *T. denitrificans* were inconclusive. They speculated about two possibilities: (i) the organism uses alternative pathways to oxidize Fe(II), involving multiple *c*-type cytochromes, or (ii) the true Fe(II) oxidase has not been identified and might be of a distinct nature.

Their targeted *c*-type cytochromes were based on a whole-genome transcriptional study, focusing on genes that were highly expressed or upregulated. If the second scenario is correct, and none of the tested proteins are involved in Fe(II) oxidation, this means that Beller et al. (2013) may have observed a general metabolic response to the absence of a viable electron donor, rather than adaptation to Fe(II) oxidation. The increased expression of *c*-type cytochromes could be related to cellular stress, such as starvation, rather than Fe(II) oxidation specifically.

The study of Beller et al. (2013), showed that denitrification genes are expressed under nitrate-reducing, Fe(II)-oxidizing conditions. However, based on the nitrite accumulation observed in our experiments, it appears that only the nitrate reductase is active. Although it is possible that the nitrite reductase is active but operates much more slowly than the upstream reduction of nitrate, the following section is based on the assumption, that the nitrite reductase is inactive. This assumption aligns with the strong fit of the applied model, where electron balance was achieved by applying the stoichiometry for biological reduction to nitrite followed by abiotic reduction to N₂O (equations 1 and 2). Full denitrification typically offers the benefit of maintaining a streamlined electron flow, which contributes to the generation of a pmf. However, the denitrifying enzymes nitrite reductase, nitrous oxide reductase, and the cytochrome *c-*dependent nitric oxide reductase (Shiro 2012), consume protons from the periplasm, thereby reducing the pmf. The absence of complete denitrification under Fe(II)-oxidizing conditions suggests that continuous electron flow may not provide a significant energy advantage. Instead, the nitrate reductase likely serves as the primary energy-conserving enzyme during nitrate-reducing Fe(II) oxidation in *T. denitrificans*. The balance between uphill (reverse electron transport) and downhill electron transport is tightly regulated in bacteria. Given that the oxidation of one Fe(II) to Fe(III) contributes only one proton to the pmf (Simon et al. 2008), it is unsurprising that metabolic regulation favors ATP production over reverse electron transport to meet the ATP demands of the cell. Even in the absence of growth, bacteria need ATP for essential processes such as membrane potential maintenance, protein synthesis, DNA repair, transport, and stress responses, all of which are critical for survival. When nitrate-reducing Fe(II) oxidation barely meets these ATP demands, energy cannot be used for reverse electron transport, which is required for generating reducing equivalents used for carbon fixation.

While this is an energetic explanation for the downhill regulation, it could also be attributed to the absence of a mechanism for Fe(III) waste management. Strict downhill regulation may be crucial because *T. denitrificans* lacks a strategy to handle Fe(III) mineralization, such as stalk formation [as observed in microaerophilic Fe(II)-oxidizers; see e.g. McAllister et al. (2019)], when relying solely on Fe(II) as an electron donor (Fig. 7B). During complete denitrification, 10 Fe(II) ions are oxidized for two nitrate molecules being reduced to one molecule of N₂ (equation 13), resulting in a Fe/N ratio of $\frac{{10}}{2} = 5$.


(13) \begin{eqnarray*}
10{\mathrm{F}}{{\mathrm{e}}^{2 + }} + 2{\mathrm{NO}}_3^ - + 24{{\mathrm{H}}_2}{\mathrm{O}} \to 10{\mathrm{Fe}}{({\mathrm{OH}})_3} + {{\mathrm{N}}_2} + 18{{\mathrm{H}}^ + }.
\end{eqnarray*}

Of the denitrifying enzymes encoded by the *T. denitrificans* genome, only the nitrate reductase contributes with two protons to pmf formation by reducing nitrate to nitrite (Beller et al. 2006a, Simon et al. 2008, Shiro 2012). If Fe(II)-derived electrons enter the quinone pool without contributing to the pmf, only the electrons utilized by nitrate reductase contribute with 1 proton per electron to the pmf. Consequently, during complete denitrification (two nitrate molecules being reduced to one molecule of N₂, equation 13), only four protons contribute to the pmf while 10 Fe(II) become oxidized. In this context, complete denitrification results in the oxidation of 2.5 Fe(II) per proton contributing to pmf. If only nitrate is reduced to nitrite (equation 1), the oxidation of each Fe(II) yields one proton and the Fe/N ratio results in $\frac{2}{1} = 2$. Since this ratio is consistent with the results of the herein presented study, it suggests that the electron transfer from Fe(II) to the quinone pool does not effectively contribute to energy conservation via the pmf, because otherwise full denitrification would be energetically beneficial and observed.

*T. denitrificans* uses the Calvin cycle for CO₂ fixation (Beller et al. 2006a,b). Fixing three CO₂ molecules into one glyceraldehyde-3-phosphate requires nine ATP and six Nicotinamide adenine dinucleotide, reduced form (NADH) (Sharkey 2019). With four protons needed per ATP synthesis and four per NADH regeneration via reverse electron transport (Simon et al. 2008, Steigmiller et al. 2008), the bacteria require 60 protons from the pmf and 12 electrons to reduce 3 CO₂ molecules. This means that 72 Fe(II) are needed to fix 3 CO₂ molecules when nitrate is reduced only to nitrite. When 60 protons are required, 60 Fe(II) become oxidized and 30 nitrate reduced, while 12 electrons derived from Fe(II) reduce three molecules of CO_2_, the Fe/N ratio results in $\frac{{72}}{{30}} = 2.4$. For complete denitrification, which may be necessary to detoxify nitrite, the required Fe(II) rises from 72 to 162. As explained by the calculation above, complete denitrification results in the oxidation of 2.5 Fe(II) per proton contributing to the pmf. When 60 protons are required, 150 Fe(II) become oxidized, 30 nitrate became reduced to 15 N_2_, while 12 electrons derived from Fe(II) reduce three molecules of CO_2_, the Fe/N ratio results in $\frac{{162}}{{30}} = 5.4$. SEM imaging results (Fig. 6) suggested that *T. denitrificans* lacks a mechanism to avoid Fe(III) accumulation on its cell surface [such as a modified cell surface charge (Saini and Chan 2013), low-pH-microenvironment (Hegler et al. 2010), or stalk formation (McAllister et al. 2019)]. If the bacterium uses reverse electron flow for CO₂ fixation despite lacking a strategy to mitigate Fe(III) encrustation, this could impede essential mass transfer with its surroundings, potentially harming cell viability. Thus, the bacterium may have evolved a regulatory mechanism that prevents uphill electron transport as a self-protection strategy. Additionally, the results of this study show that substrate utilization is incomplete and ceases abruptly, rather than gradually. Further hypothetical metabolic models incorporating this observation will be explored.

### Hypotheses explaining the inability to survive under nitrate-reducing Fe(II)-oxidizing conditions

**Figure 9.**
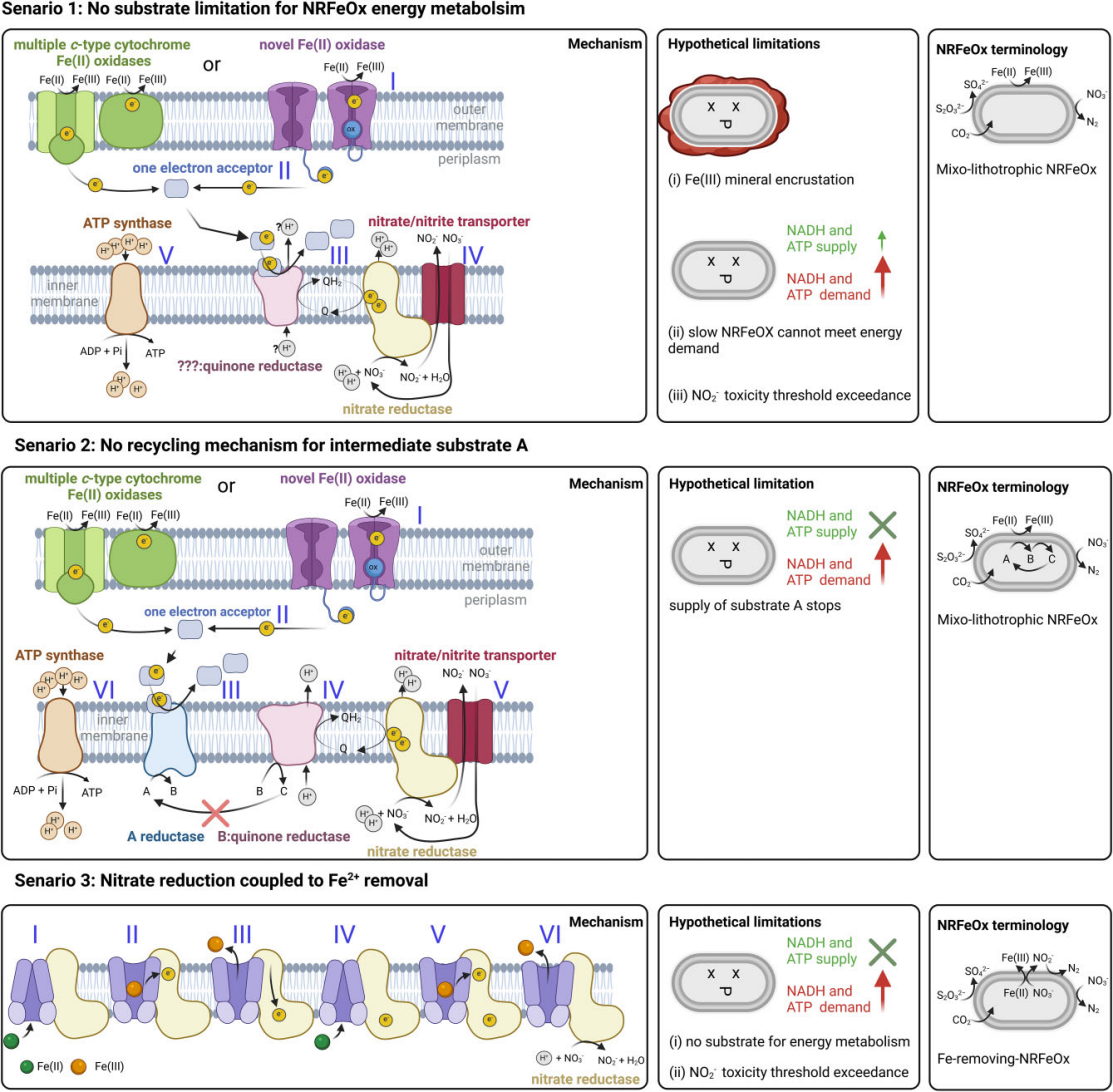
Hypothetical mechanisms responsible for incomplete nitrate-reducing Fe(II) oxidation by *T. denitrificans*. Scenario 1 considers that the process is not substrate-limited (nitrate and iron). (I) Fe(II) is oxidized by a *c*-type cytochrome Fe(II) oxidase or an unknown novel Fe(II) oxidase. (II) Electrons pass to a one-electron acceptor. (III) Two of these molecules transfer electrons to quinone reductase, reducing one quinone. (IV) Quinol: nitrate oxidoreductase reduces nitrate to nitrite, contributing to the pmf. (V) The pmf regenerates ATP. Finally, inhibition is caused by cell encrustation when Fe(III) mineralization limits mass transfer, collapsing cell homeostasis or nitrite accumulation inhibiting further nitrate-reducing Fe(II) oxidation. Scenario 2 considers dependency on a cosubstrate A. (I and II) Fe(II) oxidation proceeds as in Scenario 1. (III) Electrons are accepted by A, producing B, which (IV) is oxidized by a quinone reductase. (V and VI) Downstream reactions mirror Scenario 1. When A is unavailable, cell homeostasis collapses. Scenario 3 is a cost-efficient strategy to remove iron from inside the cell by coupling the translocation of iron to the reduction of nitrate. (I and II) Fe(II)-derived electrons are passed to the nitrate reductase, reducing its electron-accepting center. (III) The energy released is linked to the translocation of the first Fe(II) (IV and V). The second Fe(II) reduces the electron-accepting center of the nitrate reductase. (VI) Iron is translocated and both electrons collected by the reductase are transferred to nitrate, forming nitrite. Finally, the cell homeostasis collapses once ATP is depleted or nitrate-reducing Fe(II) oxidation is inhibited by nitrite accumulation. The H^+^ stoichiometry is based on Simone et al. (2008) and Steigmiller et al. (2008).

As suggested by Bryce et al. (2018), a truly autotrophic NRFeOx organism should sustain growth under nitrate-reducing Fe(II)-oxidizing conditions (Bryce et al. 2018). We transferred the initial Fe(II)/nitrate culture (transferred from thiosulfate/nitrate; i.e. first transfer) to fresh medium with nitrate and Fe(II) (second transfer), but no further cell activity was observed. Additionally, the live/dead assay at the end of the incubation of the first transfer showed mainly dead cells, and they failed to recover when transferred to medium with thiosulfate/nitrate. This indicates that the cells were adversely affected by using nitrate and Fe(II) as sole substrates. To model the inhibitory effects of nitrate-reducing Fe(II) oxidation, a noncompetitive inhibition term based on total Fe(III) concentration was applied. After 5 days, the bacterial Fe(II) oxidation rate decreased significantly, even though nitrate and Fe(II) were not fully consumed. The exact cause of this inhibition remains unclear. The following section discusses three potential scenarios that may lead to a limiting event, causing the sudden cessation of bacterial denitrification (Fig. 9). The first scenario follows a conventional approach (Carlson et al. 2013, Becker et al. 2021), while the other two are primarily introduced here. However, all three scenarios are hypothetical and equally speculative. Although additional scenarios could be proposed, the two unconventional ones presented here are meant to inspire broader thinking within the NRFeOx research community.

***Scenario 1:*** “No-substrate-limitation” for NRFeOx energy metabolism: nitrate-reducing Fe(II) oxidation stops either due to limited mass transfer caused by Fe(III) mineralization around the cells, or because constantly increasing nitrite concentrations reach a specific toxic threshold value and nitrite becomes inhibiting for the denitrification activity.
***Scenario 2:*** “No-recycling-mechanism” for an intermediate substrate: the reaction halts as the cosubstrate becomes depleted.
***Scenario 3:*** Nitrate reduction coupled to Fe^2+^ removal: nitrate reduction is not coupled to energy production, so either it ceases when the cell’s energy reserves are exhausted, or because constantly increasing nitrite concentrations reach a specific toxic threshold value and nitrite becomes inhibiting for the denitrification activity.

Scenario 1 represents a very conventional model, aligning with commonly proposed nitrate-reducing Fe(II) oxidation pathways. In this pathway, the Fe(II) oxidase reduces an electron acceptor molecule, which then serves as the substrate for a quinone reductase. The quinone, in turn, becomes the substrate for the nitrate reductase. Referring to the final substrate concentrations in the experiment, this pathway shows no shortage of substrates. It is reasonable to assume that the observed inhibition is due to cell encrustation, as has been observed multiple times under conditions involving nitrite and Fe(II) at neutral pH (Emerson and Moyer 1997, Kappler et al. 2005, Miot et al. 2009, Schädler et al. 2009, Cheng et al. 2022). Further the level of encrustation overserved in this study (Fig. 7B) has been comparable to the cell mineral encrustation observed for autotrophic culture KS, cultivated with 10 mM Fe(II) and 4 mM nitrate after 4 days (Huang et al. 2023). This raises the question: to what extent does Fe(III) mineral encrustation inhibit NRFeOx? Are there qualitative differences in the encrustation observed in autotrophs compared to other NRFeOx systems? For example, could the porosity or location of encrustation (outer surface versus periplasm) play a role, which might not be fully distinguishable by commonly used microscopy techniques? Moreover, does encrustation lead to a rapid decline in bacterial activity, or does it cause a more gradual decrease proportional to the level of encrustation, affecting the metabolite exchange between the cell and its environment? If encrustation is indeed the cause of inhibition, it suggests that this process remains harmless until a threshold is reached for mass transfer, after which the cell’s homeostasis collapses. This implies that a certain ATP demand can no longer be met once mass transfer falls below this threshold, leading to cell death.

Another explanation could be that the conditions in the microenvironment at the cell surface differs significantly from the conditions in the bulk culture (and as a consequence, leads to wrong conclusions if only considering the bulk geochemical data). Specifically, the concentration of Fe(III) (oxyhydr)oxide around the cell is very high due to cell mineral incrustation (Fig. 7B). This is detrimental because aqueous Fe(II) is adsorbed in the proximity of the cell, making it less mobile and it is possibly no longer bioavailable. The result is a sink of aqueous Fe(II) at the cell surface. In addition, the ratio of Fe(II) to the precipitated iron (oxyhydr)oxide may be lower in this microenvironment of the cell, leading to much faster abiotic oxidation rates than observed for the whole system (Tai and Dempsey 2009). This is likely to be further enhanced by the local accumulation of NO_2_^−^ caused by cell excretion. As a result, Fe(II) in the microenvironment of the encrusted cell is no longer available to the bacteria, and the concentration of aqueous Fe(II) may be so low that biological denitrification is thermodynamically infeasible.

Another cause of inhibition could be a toxic effect due to the nitrite accumulation of up to 1.65 mM observed here. This is a likely scenario because impaired microbial activity due to nitrite has been observed for several *T. denitrificans* strains (Baalsrud and Baalsrud 1954, Baldensperger and Garcia 1975, Claus and Kutzner 1985). Claus and Kutzner (1985) describe, for a different *T. denitrificans* strain using thiosulfate as an electron donor, no inhibitory effect of nitrite on denitrification for nitrite concentrations of up to 2 mM and a strong inhibitory effect at a nitrite concentration of 6.5 mM (Claus and Kutzner 1985). Similarly, nitrite could be a possible cause for the observed incomplete nitrate reduction in the here presented Fe(II)-oxidizing culture of *T. denitrificans* (ATCC 25259). After a latency phase of about 1 day, the nitrate-reducing Fe(II) oxidation proceeds at approximately maximum speed until about 4.5 days (Fig. 7), with a denitrification rate (r_biotic_) of $0.27\,\,\frac{{mM}}{d}\,\,$to $0.26\,\,\frac{{mM}}{d}$, and the nitrite concentration measured after 5 days is 1.11 mM. Thus, a nitrite concentration starting at about 1 mM may represent a potential threshold for nitrite toxicity for *T. denitrificans* (ATCC 25259) incubated with 10 mM Fe(II) and 3.5 mM nitrate.

Another study conducted by D’Aquino et al. (2023) on *T. denitrificans* (DSM 12475), investigating the effects of inorganic ions on both autotrophic and heterotrophic denitrification, observed an intermediate nitrite accumulation of up to ~21 mM, which was almost completely consumed (∼95%) by the bacteria during the course of the experiment. One of the heterotrophic setups was performed in the presence of 7.20 mM Fe(II), and all autotrophic setups contained 19.20 mM thiosulfate and 7.20 mM Fe(II) (D’Aquino et al. 2023). Thus, this strain showed sufficient activity at nitrite concentrations of up to 20 mM and was able to remove nitrite under the conditions tested, which contained, in addition to Fe(II), also organic substrates (yeast extract) or thiosulfate as electron donors.

Scenario 2 assumes that the availability of a necessary substrate or cofactor becomes a limiting factor, leading to rapid inhibition. While scenario 2 is unconventional, it is no less speculative than scenario 1. Pinpointing specific explanations for this model is complex, as numerous hypothetical pathways involving substrate limitation may exist, beyond the basic model shown in Fig. 9. In this model, electrons derived from Fe(II) reduce substrate A to B, which then donates electrons to the quinone pool by conversion to C. If the reducing power is lacking, C cannot be recycled back to A. Substrate A could be a membrane-bound protein that receives electrons from Fe(II) and reduces an intermediate of the tricarboxylic acid cycle, such as malate, acting similarly to malate oxidoreductase (MQO) (Kabashima et al. 2013), but with a specialized subunit to receive electrons from an intermediate reduced by Fe(II). Malate could then be converted to pyruvate, and pyruvate to formate, with formate functioning as substrate C, which reduces the quinone pool. In this scenario, the quinone reductase could be formate dehydrogenase. Nitrate reductase often forms a respiratory chain with formate dehydrogenase (Bertero et al. 2005), and electron transfer from formate to nitrate is coupled with proton translocation across the inner membrane, generating a pmf via a redox loop mechanism (Mitchell 1976).

An alternative, though more complex scenario, which is not represented by the model in Fig. 9, involves the primary electron acceptor lacking sufficient reducing potential to reduce quinone (which requires two electrons per quinone). However, in combination with an electron from another substrate (or cofactor) with slightly higher reducing potential, it may become thermodynamically favorable to use both electrons. In such cases, a protein might receive one electron from Fe(II) oxidase and one from the additional substrate to reduce quinone. As maintaining these processes is essential, the cell would spend its stored reducing power to regenerate this substrate until the reserve is depleted. This raises the question of where the second electron from Fe(II) goes during nitrate reduction, given that the Fe(II)_oxidized_/nitrate_reduced_ ratio would be 1 versus the observed ratio of 2. One possible explanation is that an additional reaction, involving electron bifurcation, loses one electron, which is used to reduce a second additional substrate.

In the case of Scenario 3, Fe(II) oxidation is considered to be a stress response and part of a strategy to efficiently remove Fe(II) from the bacterial cell while simultaneously reducing nitrate to nitrite. Beller et al. (2013) identified upregulated genes in *T. denitrificans* when incubated with both iron(II) and nitrate, which may contribute to Fe(II) translocation. Based on bioinformatic analysis using AlphaFold Protein Structure Database, UniProt, Foldseek, and STRING the below hypothetical functions where assigned to proteins encoded by the *T. denitrificans* genome (Beller et al. 2006a, Szklarczyk et al. 2023, The UniProt Consortium 2023, van Kempen et al. 2024, Varadi et al. 2024). Specifically, two of the genes found upregulated, i.e. Tbd_1320 and its neighboring gene Tbd_1321 may be involved in denitrification processes, while Tbd_1323 has similarities to nitrogen fixation proteins. Downstream of Tbd_1320, two genes encode a multicopper oxidase, *pcoAB* (Tbd_1324 and Tbd_1325), which are believed to contribute to copper resistance (Mellano and Cooksey 1988). Based on findings by Huston et al. (2002), which demonstrated that PcoA homologues in *Pseudomonas aeruginosa* function as ferroxidases involved in iron acquisition, He et al. (2016) hypothesized that these homologues could play a role in Fe(II) oxidation within nitrate-reducing Fe(II) oxidation metabolism (Huston et al. 2002, He et al. 2016). Further, these proteins could potentially interact with those encoded by Tbd_2741, also identified as an upregulated gene, whose protein structure shows similarities to membrane-bound electron transport proteins. Additionally, Tbd_2740 encodes a heavy metal-translocating P-type ATPase, and Tbd_2742 encodes a ferredoxin, further suggesting a role in electron transfer during these processes.

The nitrite accumulation observed in our experiments could support our hypothesis that an alternative nitrate reductase, distinct from the membrane-bound nitrate reductase Nar, may act as the primary nitrate acceptor, for the following reasons. Nitrite is known to have toxic effects on *T. denitrificans* (Baalsrud and Baalsrud 1954, Baldensperger and Garcia 1975, Claus and Kutzner 1985), and since *T. denitrificans* has the genomic capacity to metabolize nitrite, one would expect the cells to prevent its accumulation. However, since nitrite accumulation was evident in the here presented study, two possibilities arise: (i) nitrite at a concentration of up to 1.65 mM does not exert toxic effects on the cells and therefore does not necessitate detoxification. (ii) *T. denitrificans* is unable to metabolize nitrite using electrons derived from Fe(II) oxidation. The second scenario implies that the nitrate-reducing Fe(II) oxidation pathway does not involve reduction of the quinone pool. While Tian et al (2024) argued that Fe(II) has a sufficiently negative reduction potential to reduce the quinone pool, our results may indicate otherwise. Assuming that the quinone is not reduced by electrons stemming from Fe(II) oxidation in the system presented here, the cytochrome *c* reductase (cytochrome *bc*_1_ complex) would not be able to regenerate reduced cytochrome *c*, the electron donor required by the nitrite reductase, which would result in the observed accumulation of nitrite (for the denitrification cascade see Fig. 8, expected hypothetical mechanism).

Thus, the accumulation of nitrite suggests that Fe(II) oxidation by *T. denitrificans* cannot regenerate the quinone pool. Consequently, the nitrate-reducing enzyme must be one that operates independently of the quinone pool, since the membrane-bound nitrate reductase Nar depends on quinol as a substrate. Therefore, this finding is consistent with the proposed scenario 3, which postulates the involvement of an alternative nitrate reductase.

Furthermore, the inability to couple Fe(II) oxidation to quinone reduction negates the possibility of reverse electron transport to generate reducing power for autotrophic growth. This inability challenges the traditional autotrophic paradigm in nitrate-reducing Fe(II) oxidation, as discussed in the section “Challenging the autotrophic paradigm in nitrate-reducing Fe(II) oxidation.”

### Reevaluating the classification of NRFeOx: beyond traditional autotrophic and mixotrophic lifestyles

In the literature, NRFeOx are either categorized as autotrophs or heterotrophs. The latter is commonly referred to as mixotrophic, as the chemolithotrophic pathway of nitrate-reducing Fe(II) oxidation runs in parallel to a chemoorganotrophic pathway. These two terms refer to the energy-yielding mechanisms, but it remains complicated to estimate the contributions of the energy yield from each type of substrate. While it involves a mixture of using both inorganic and organic substrates, the corresponding bacteria are facultative mixotrophs.

*Thiobacillus denitrificans* cannot be categorized in any of these two groups. *Thiobacillus denitrificans* is an autotroph when using thiosulfate (inorganic) as its sole source of electrons. Calling *T. denitrificans* an autotrophic NRFeOx is not necessarily incorrect, as it is an autotroph and a NRFeOx. However, this designation is misleading as it may imply that it grows autotrophically under nitrate-reducing Fe(II)-oxidizing conditions which is not the case. Since there are truly autotrophic NRFeOx enrichment cultures that grow autotrophically oxidizing Fe(II) for both nitrate reduction and CO_2_ fixation (Straub et al. 1996, Jakus et al. 2021, Huang et al. 2023), it is not recommended to assign a microorganism as an autotrophic NRFeOx if the autotrophy does not refer to using Fe(II) both as an electron donor for nitrate reduction and as an electron donor for fixing inorganic carbon. It might be clearer to refer to it as a non-autotrophic NRFeOx, additionally mentioning that it can grow autotrophically with other inorganic substrates (e.g. thiosulfate). If *T. denitrificans* is further investigated and evidence supports either scenario 1 or 2, it could be classified as a mixo-lithotrophic NRFeOx, as both thiosulfate and iron(II) are inorganic electron donors. If scenario 3 is supported, it might be more appropriately termed a Fe(II)-removing NRFeOx or Fe(II)-stress-related NRFeOx, reflecting its role in managing Fe(II) stress rather than energy conservation.

## Conclusions and outlook

*Thiobacillus denitrificans* does not exhibit chemolithoautotrophic growth under nitrate-reducing, Fe(II)-oxidizing conditions, and it fails to sustain nitrate-reducing Fe(II) oxidation over multiple transfers. This indicates that while the strain can carry out nitrate-reducing Fe(II) oxidation, it does not concurrently conduct autotrophy. The inability to survive long-term under these conditions and the higher mortality compared to nutrient-starvation scenarios (represented by controls missing one or both substrates) suggest that in this strain, nitrate-reducing Fe(II) oxidation does not support growth on its own. However, it may offer an energy advantage when combined with a growth-supporting electron donor. In the context of microbial flexibility, negative outcomes, like those reported here, are crucial for uncovering knowledge gaps and questioning established assumptions. Although *T. denitrificans* can utilize Fe(II) as an electron donor, its use seems to be directed primarily toward energy conservation rather than growth, even though the latter might theoretically be possible. This paradox remains a key focus for future research.

The study emphasizes a core issue in the classification of NRFeOx organisms. Our study has shown that *T. denitrificans* is a non-autotrophic NRFeOx, but further research is required to determine whether it is mixo-lithotrophic (scenarios 1 and 2) or functions as an Fe(II)-removing NRFeOx (scenario 3).

If *T. denitrificans* is indeed mixo-lithotrophic, its environmental role in nitrate-reducing Fe(II) oxidation should be explored further. *Thiobacillus denitrificans*’ ability to oxidize pyrite (FeS₂) for denitrification suggests that nitrate-reducing Fe(II) oxidation, coupled with sulfur species oxidation, might support an autotrophic lifestyle (Torrentó et al. 2010). However, if Fe(II) oxidation is merely a stress response to intracellular Fe(II) toxicity, its environmental role may be more limited, functioning as a survival strategy rather than contributing to energy conservation.

To confirm whether *T. denitrificans* is mixo-lithotrophic, ATP measurements should be conducted to demonstrate energy production. Alternatively, structural studies of hypothetical proteins involved in Fe(II) transport and proteins standing in genomic relationship with hypothetical nitrate-binding domains could provide further insights. In the near future, the rapid advancements in bioinformatics tools driven by artificial intelligence may allow researchers to revisit foundational studies, such as those by Beller et al. (2013). Unknown proteins identified in transcriptomic studies, alongside their genomic neighbors or other genes potentially linked to a common function, might soon be placed in a broader context, helping us better understand this process and align it with the herein proposed scenarios.

## Supplementary Material

fiaf024_Supplemental_File
