# Supplementary material for: Evaluation of Thiobacillus denitrificans’ sustainability in nitrate-reducing Fe(II) oxidation and the potential significance of Fe(II) as a growth-supporting reductant
Source: FEMS Microbiol Ecol. 2025 Mar 17;101(4):fiaf024. doi: 10.1093/femsec/fiaf024 (PMC11963766; doi:10.1093/femsec/fiaf024)
Supplement: fiaf024_Supplemental_File [file fiaf024_supplemental_file.docx]

**SUPPLEMENTAL INFORMATION**

**Evaluation of *Thiobacillus denitrificans*' sustainability in nitrate-reducing Fe(II) oxidation and the potential significance of Fe(II) as a growth-supporting reductant**

*Stefanie Becker^1*^, Thu Trang Dang^1^, Ran Wei^2^, Andreas Kappler^1,3*^*

^1^Geomicrobiology, Department of Geosciences, University of Tübingen, Schnarrenbergstrasse 94-96, D-72076 Tübingen, Germany

^2^Institute for Modelling Hydraulic and Environmental Systems (IWS), Department of Stochastic Simulation and Safety Research for Hydrosystems, University of Stuttgart, Pfaffenwaldring 5a,70569 Stuttgart, Germany

^3^Cluster of Excellence: EXC 2124: Controlling Microbes to Fight Infection, Tübingen, Germany

^*^To whom correspondence should be sent:

**Stefanie Becker**, Geomicrobiology, Department of Geosciences

University of Tuebingen, Schnarrenbergstrasse 94-96, D-72076 Tuebingen, Germany

Phone: +49-7071-2974690; Email: stefanie.becker@geo.uni-tuebingen.de

**Andreas Kappler**, Geomicrobiology, Department of Geosciences

University of Tuebingen, Schnarrenbergstrasse 94-96, D-72076 Tuebingen, Germany

Phone: +49-7071-2974992; Email: [andreas.kappler@uni-tuebingen.de](mailto:andreas.kappler@uni-tuebingen.de)

**Supplemental information - Fe sample collection and terminology**

**
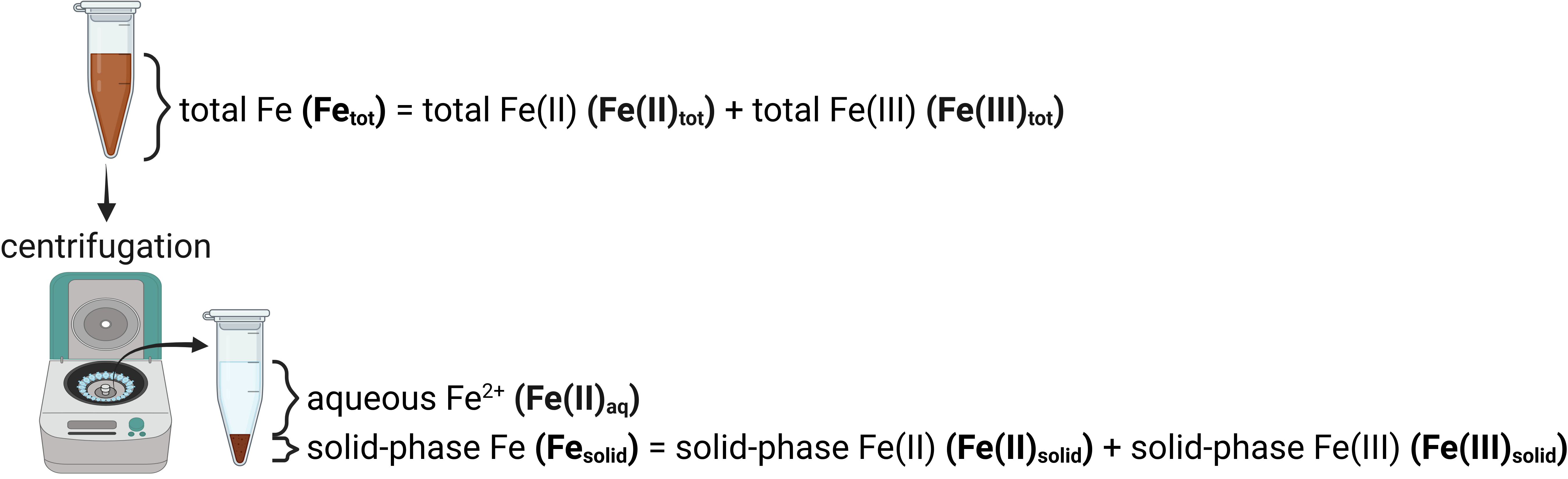
**

Figure S1: Sampling fractions of total Fe, aqueous Fe^2+^ and solid-phase Fe. In bold are the respective abbreviations, which are commonly used in the literature and also in the main text of the presented study.

**Supplemental information - additional data sets**


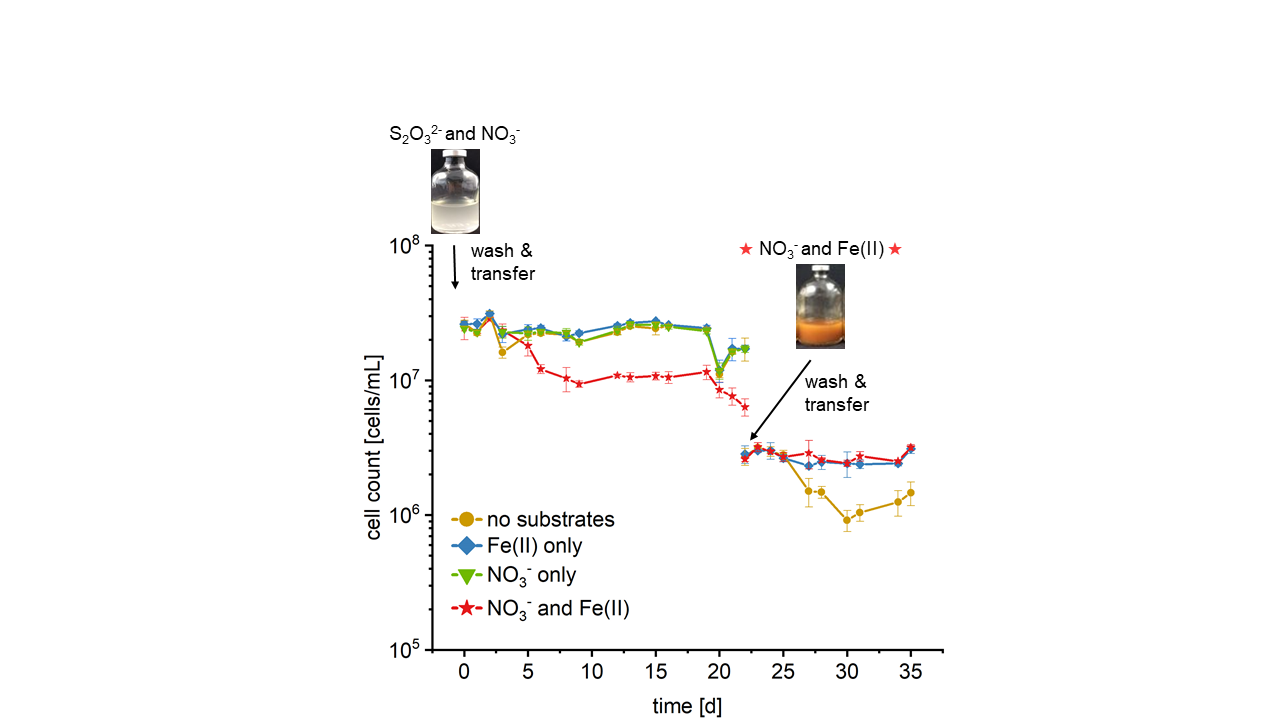


Figure S2: Cell number over time in setups containing 10 mM Fe(II), 4 mM nitrate, and T. denitrificans compared to cultures lacking either nitrate, Fe(II) or both. Please note that at day 22, the NO_3_^-^/Fe(II) culture was centrifuged, washed and transferred onto fresh medium with either NO_3_^-^/Fe(II), Fe(II) only or none of both substrates. Error bars represent the standard deviation of biological replicates (3-5 replicates, Fig. 1 main text). Absence of error bars indicate error was smaller than symbol size.


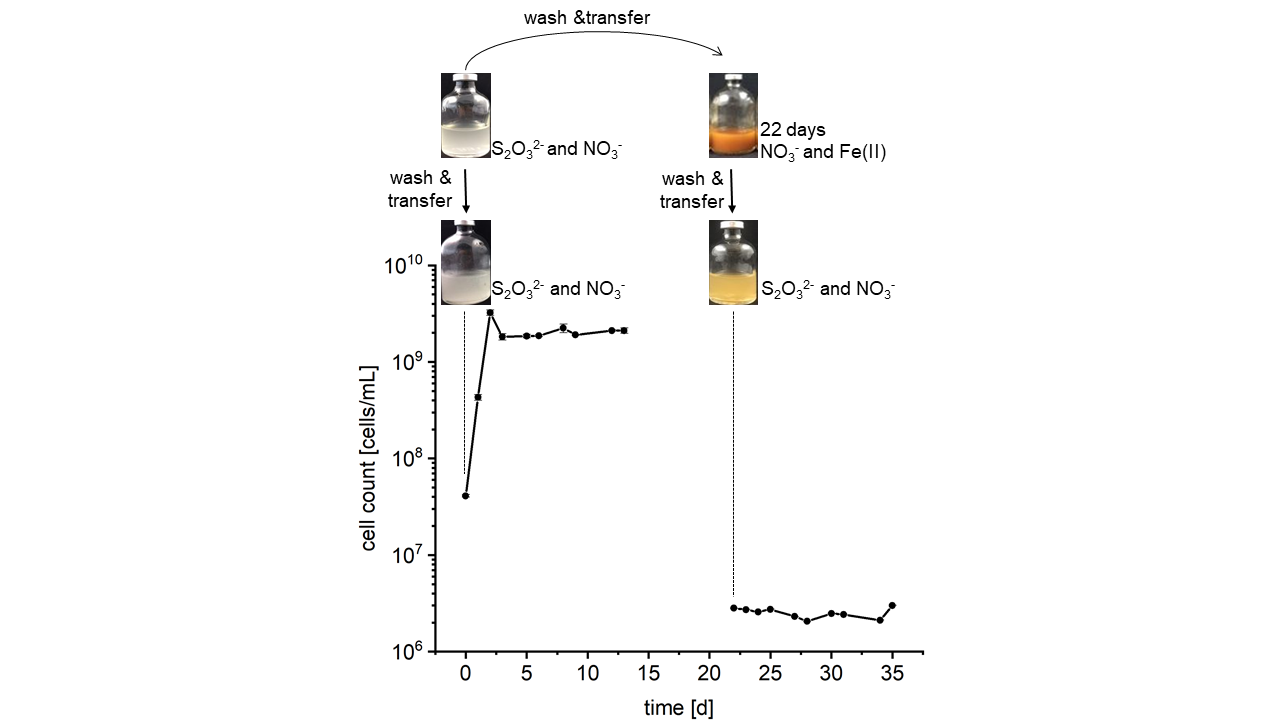


Figure S3: Cell counts in cultures with thiosulfate and nitrate as substrates to determine the viability of T. denitrificans over the course of an experiment to evaluate its ability to continuously perform autotrophic nitrate-reducing Fe(II) oxidation. The thiosulfate/nitrate cultures were inoculated using the same T. denitrificans suspension as used in the main experiment. The inoculum for the 1^st^ transfer (days 0-14) was harvested from a thiosulfate/denitrifying culture at the late exponential phase. The same T. denitrificans suspension was used to inoculate medium containing Fe(II)/nitrate. For the 2^nd^ transfer on day 22, this nitrate-reducing and Fe(II)-oxidizing culture was used to inoculate further experimental setups and those was evaluated for its viability by medium containing thiosulfate/nitrate again (days 22-35). The error bars correspond to three technical triplicates.


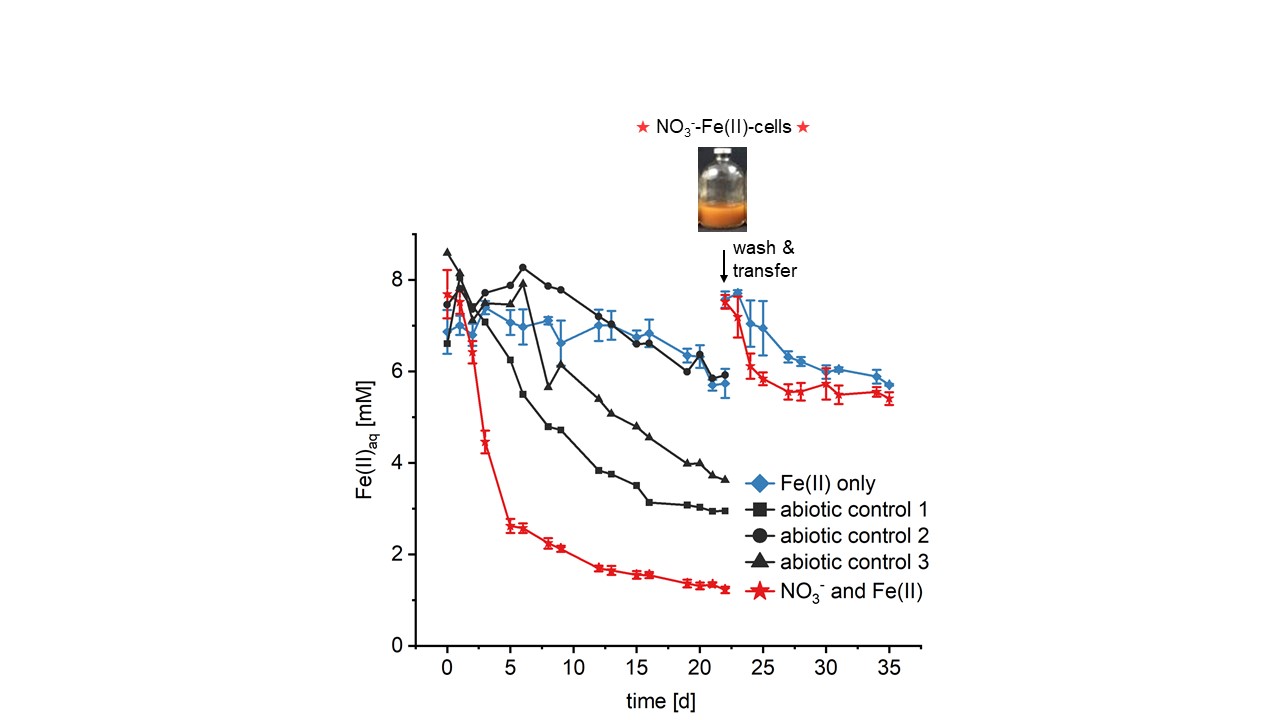


Figure S4: Decrease of aqueous Fe(II) (Fe^2+^_aq_) due to precipitation of Fe(II) minerals and oxidation in cultures containing 10 mM Fe(II), 4 mM nitrate, and T. denitrificans (red), Fe(II) and T. denitrificans (blue), and in three abiotic controls containing Fe(II) and nitrate (black), where the loss of iron is solely due to abiotic processes such as Fe(II) mineral precipitation and Fe(II) adsorption to the glass wall of the serum bottle. Please note that at day 22, the NO_3_^-^/Fe(II) culture was centrifuged, washed and transferred onto fresh growth medium containing either NO_3_^-^/Fe(II) or Fe(II) only. Error bars represent the standard deviation of biological replicates (3-5 replicates, Fig. 1 main text).

**Supplemental information on Fe(III) calculation**

Just before sampling, the serum bottles were shaken to obtain a homogeneous distribution of solid-phase iron. Because of fast mineral particle sedimentation, this procedure has its limitations and slight handling differences can result in less total iron in the taken sample than initially added to the medium, causing fluctuations in total iron concentration analyses. Some iron may also associate with the cell wall via sorption and/or precipitation. When calculating Fe(III) by subtracting Fe(II) from the total Fe, it reflects the Fe(III) concentration in the sample taken, not in the entire serum bottle. By calculating the ratio of Fe(III) to Fe(tot) in the sample and multiplying this ratio by 10 mM, we can estimate the total Fe(III) concentration in the culture. This method assumes that the ratio of Fe(III) to Fe(tot) in the sample is the same as in the entire culture (including iron adsorbed on glass wall). Consequently, this second calculation method leads to slightly higher Fe(III) concentrations (Fig. S5). When applying a model calibrated with this data, it is easier to consider the concentration of the entire culture, even though it may be slightly biased.


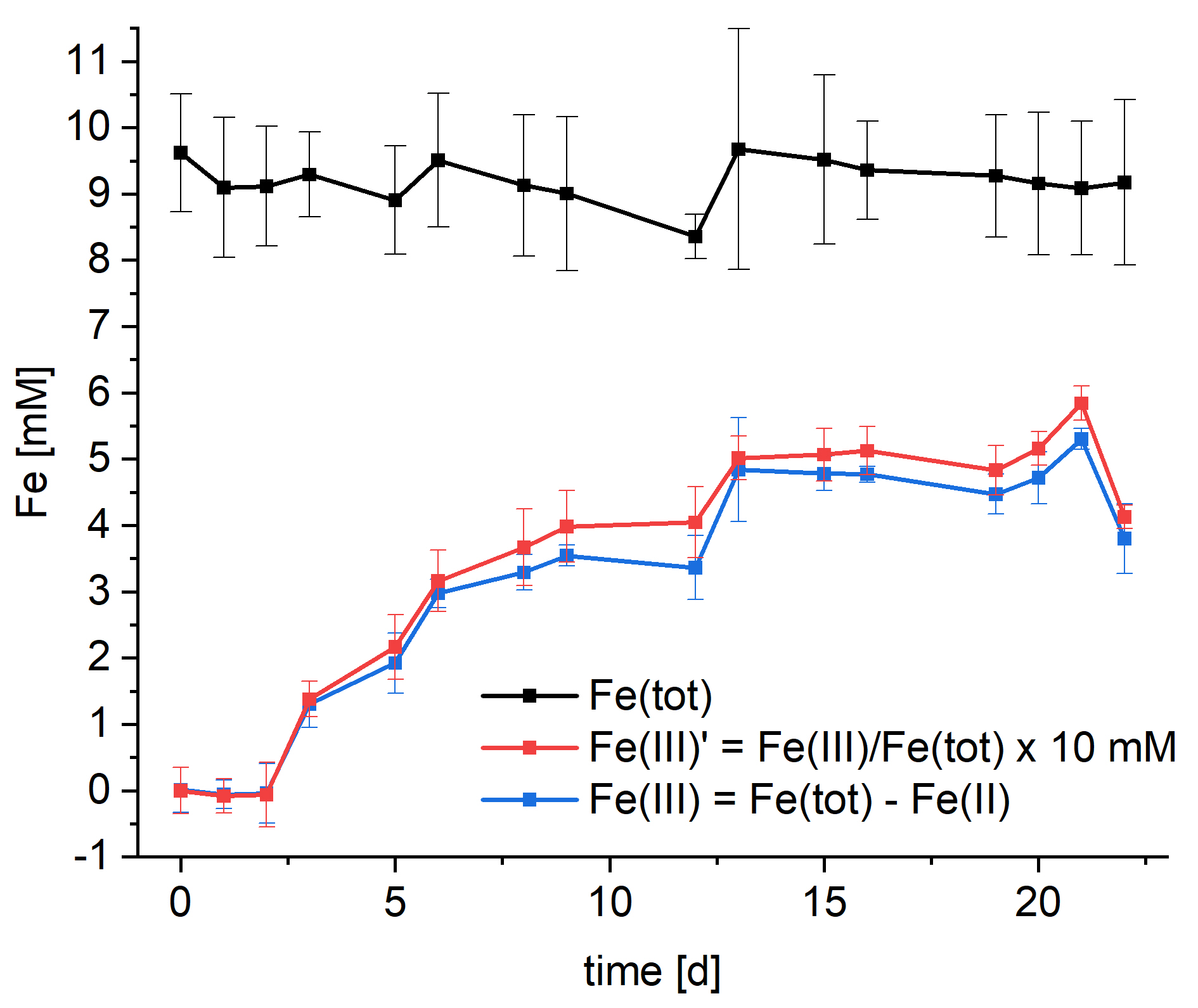


Figure S5: Fe(III) concentrations of nitrate-reducing Fe(II)-oxidizing T. denitrificans culture (initial substrate concentration of 10 mM Fe(II) and 4 mM nitrate), based on different calculation strategies and the corresponding measured Fe(tot) concentrations. Error bars indicate the standard deviation of biological replicates. Fe(tot) are solid and dissolved Fe(II) and Fe(III). Fe(II) are solid and dissolved Fe(II). The iron samples were diluted in in SA:HCl (40 mM amidosulfonic acid, 1 M HCl) subsequently to collection (Granger & Sigman, 2009; Klueglein & Kappler, 2013) and Fe(tot) (black data) and Fe(II) were quantified by a colorimetric ferrozine-based assay (Schaedler et al., 2018; Stookey, 1970). Fe(III) was reduced to Fe(II) using HAHCl (Hydroxylammonium hydrochloride 10% w/v, 1 M HCl), therefore both Fe(tot) and Fe(II) were determined as ferrozine–Fe(II) complex. Fe(II) and Fe(III)(red and blue data) in % was calculated using the results from Fe(II) and Fe(tot). Fe(III)’ (red data) was calculated by: (Fe(III))/(Fe(tot))×10 mM , where 10 mM is the initial Fe(II) concentration. Fe(III) (blue data) is the difference of Fe(tot) and Fe(II).

**Supplemental information on the kinetic model for the simulation of biotic and abiotic denitrification coupled to Fe(II) oxidation**

Abiotic denitrification can occur when NO₂⁻ reacts with Fe²⁺ in solution (a homogeneous reaction), leading to Fe(III) mineral precipitation. Additionally, NO₂⁻ can react with both Fe²⁺ in solution and Fe(II) adsorbed onto solid Fe(III) surfaces (a heterogeneous reaction). Since the latter reaction is faster, abiotic denitrification involving NO₂⁻ and Fe(II) is autocatalyzed (Jones et al. 2015). Additionally, Fe(II) can bind to cell surfaces, where it reacts with NO_2_^-^ (Coby & Picardal 2005). Regardless of the origin, Fe(III) hydroxides, oxyhydroxides, or oxides bind Fe(II) and function as catalysts. Consequently, the presence of bacterial cells and bacterial Fe(II) oxidation reinforces abiotic Fe(II) oxidation. The model simplifies these mechanisms by: (1) treating all Fe(II) species the same (Fe^2+^, Fe(II)-cell bound, Fe(II) adsorbed to Fe(III), Fe(II) minerals), (2) assuming all metabolized Fe(II) precipitates as Fe(III) at the cell surface, (3) which then rapidly adsorbs Fe(II) to cell-bound Fe(III), so the adsorption rate does not limit the rate of abiotic Fe(II) oxidation, (4) implying the positive effect of Fe(III) (heterogenous reaction), by having the abiotic denitrification rate follow first-order kinetics with respect to [Fe(III)] but not Fe(II)_solid-bound_ and (5) assuming that all abiotic Fe(II) oxidation takes place near the cell, causing further encrustation and leading to greater inhibition, by having the inhibition term dependent on total [FeIII] (product of abiotic and biotic Fe(II) oxidation). As the calculated concentrations using the calibrated model align well with the observed concentrations, these simplifications are effective for the presented system.

**Toxicity function**

A variation of commonly used toxicity functions to simulate non-competitive inhibition where applied, in order to evaluate the best mathematical function describing irreversible product inhibition by cell encrustation, these include:

- First order inhibition function (Poinapen & Ekama 2010)

$f_{tox}=1-\frac{[Fe\left( III \right)tot]}{{IC}_{50}}$ (SI1)

- Monod-type inhibition function (Poinapen & Ekama 2010; Robles et al. 2020):

$f_{tox}=\frac{{IC}_{50}}{{IC}_{50}+[Fe\left( III \right)tot]}$ (SI2)

- Two-parameter logistic inhibition function (Kalyuzhnyi et al. 1998)

$f_{tox}=\frac{1}{1+\left( \frac{\left[ Fe(III) \right]}{{IC}_{50}} \right)^{\frac{log(99)}{log(\frac{K_{100}}{{IC}_{50}})}}}$ (SI3)

When $\frac{log(99)}{log(\frac{K_{100}}{{IC}_{50}})}=\rho$the equation can be expressed as (Belli et al. 2015):

$f_{tox}=\frac{1}{1+\left( \frac{\left[ Fe(III) \right]}{{IC}_{50}} \right)^{\rho}}$ (SI4)

- Exponential inhibition function (Poinapen & Ekama 2010)

$f_{tox}=e^{{-(\frac{\left[ Fe\left( III \right) \right]}{1.20112\times{IC}_{50}})}^{2}}$ (SI5)

Where, K100 denote for the concentrations of Fe(III) corresponding to a hundredfold decrease in bacterial denitrification rate and IC_50_ is an inhibitory constant representing the Fe(III) concentration at which cell encrustation causes 50% (twofold decrease) of inhibition. The toxicity exponent $\rho$ or $\rho'$ determines the slope around the inflection point of 50% inhibition. The slope of the toxicity terms is shown in figure S6.

**Abiotic Fe(II) oxidation by nitrite**

It has been observed that Fe(II) reacts in presence of Fe(III) minerals with nitrite faster than aqueous Fe^2+^_aq_. In order to evaluate the impact of adsorption of Fe^2+^_aq_ on Fe(III) minerals (equation 3) (Tai and Dempsey, 2009) on the abiotic denitrification rate of the presented system, the simulation was run with rate functions dependent and independent of total Fe(II) or Fe(III) concentration, respectively. Further, the abiotic reaction was evaluated for first order kinetic vs multi-molecular kinetic in respect to the substrates. The equations (SI6), (SI7) and (SI8) follow first order kinetics for Fe(II), Fe(III) and NO_2_^-^ respectively.

$r_{abtiotic}=k_{\mathrm{abtiotic}} {[Fe(II)][Fe(III)]\left[ \mathrm{NO}_{2}^{-} \right]}$ (SI6)

$r_{abtiotic}=k_{\mathrm{abtiotic}} {[Fe(II)]\left[ \mathrm{NO}_{2}^{-} \right]}$ (SI7)

$r_{abtiotic}=k_{\mathrm{abtiotic}} {[Fe(III)]\left[ \mathrm{NO}_{2}^{-} \right]}$ (SI8)

Where, k_abiotic_ is the kinetic constant of the abiotic rate. The equation (SI9), (SI10) and (SI11) represent multi-molecular kinetic rate equations.

$r_{abtiotic}=k_{\mathrm{abtiotic}} \left[ Fe(II) \right]^{4}\left[ Fe(III) \right]^{4} \left[ \mathrm{NO}_{2}^{-} \right]^{2}$ (SI9)

$r_{abtiotic}=k_{\mathrm{abtiotic}} \left[ Fe(II) \right]^{4} \left[ \mathrm{NO}_{2}^{-} \right]^{2}$ (SI10)

$r_{abtiotic}=k_{\mathrm{abtiotic}} \left[ Fe(III) \right]^{4} \left[ \mathrm{NO}_{2}^{-} \right]^{2}$ (SI11)

**Evaluation of the kinetic models**

The NRMSE values for each model variant are detailed in table S1. The inhibitory effect of the toxicity terms simulating cell-encrustation (equation 5-8) are shown in figure S6. The aim was to determine the model that best captures the dynamics of the abiotic reaction during the final 9 days of the experiment, where it nearly equals bacterial denitrification and subsequently becomes dominant. Given the smaller technical error associated with nitrite measurements compared to Fe(II), we identified the model with the lowest NRMSE regarding nitrite data during this crucial period as the most suitable fit. In this context, the two-parameter logistic inhibition function (equation 7) emerged as the optimal toxicity term for describing the inhibitory effect of Fe(III) encrustation, while the abiotic reaction was best represented by a rate function that is first order with respect to Fe(II), Fe(III), and nitrate (equation 9).


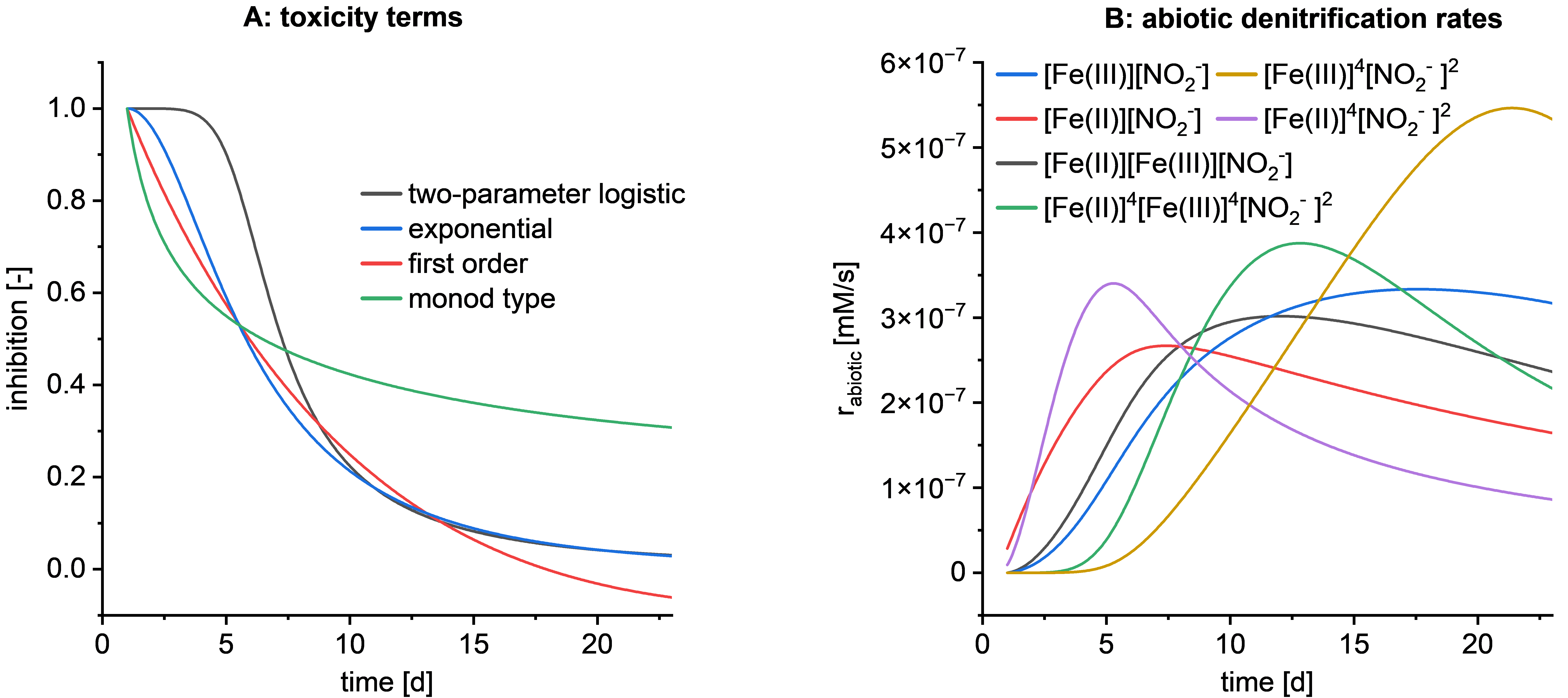


Figure S6: A. Toxicity terms: Inhibition effect of various inhibition functions (equation 5-8) utilized to determine the most suitable model for representing the toxicity of Fe(III) cell encrustation .B. Abiotic denitrification rates: Estimated abiotic denitrification based on functions applying either first order or multi molecular kinetics. The functions further differ in their substrate dependency. $r_{abtiotic}=k_{abtiotic} \left[ x \right],$where x stands for one of the variants given at the figure’s labels.

Table S1: NRMSE of the simulation using different toxicity functions.

| **Name** | **Function / term** | **NRMSE_overall_** | | **NRMSE_last9days_** | |
| --- | --- | --- | --- | --- | --- |
| **First order**  (Poinapen & Ekama 2010) | $f_{tox}=1-\frac{[Fe\left( III \right)tot]}{{IC}_{50}}$ | NO_3_^-^ | 0.044 | NO_3_^-^ | 0.209 |
|  |  | NO_2_^-^ | 0.054 | NO_2_^-^ | 0.219 |
|  |  | Fe(II) | 0.063 | Fe(II) | 0.828 |
| **Monod-type**  (Poinapen & Ekama 2010; Robles et al. 2020): | $f_{tox}=\frac{{IC}_{50}}{{IC}_{50}+[Fe\left( III \right)tot]}$ | NO_3_^-^ | 0.070 | NO_3_^-^ | 0.830 |
|  |  | NO_2_^-^ | 0.074 | NO_2_^-^ | 0.179 |
|  |  | Fe(II) | 0.088 | Fe(II) | 1.504 |
| **Two-parameter logistic**  (Kalyuzhnyi et al. 1998) (Belli et al. 2015) | $f_{tox}=\frac{1}{1+\left( \frac{\left[ Fe(III) \right]}{{IC}_{50}} \right)^{\rho}}$ | NO_3_^-^ | 0.038 | NO_3_^-^ | 0.275 |
|  |  | NO_2_^-^ | 0.034 | NO_2_^-^ | 0.069 |
|  |  | Fe(II) | 0.069 | Fe(II) | 0.919 |
| **Exponential**  (Poinapen & Ekama 2010) | $f_{tox}=e^{{-(\frac{\left[ Fe\left( III \right) \right]}{1.20112\times{IC}_{50}})}^{2}}$ | NO_3_^-^ | 0.044 | NO_3_^-^ | 0.320 |
|  |  | NO_2_^-^ | 0.047 | NO_2_^-^ | 0.086 |
|  |  | Fe(II) | 0.071 | Fe(II) | 1.070 |

Table S2: NRMSE of simulation using different rates for the change of Fe(III) by abiotic denitrification.

| **Name** | **Rate function** | **NRMSE_overall_** | | **NRMSE_last9days_** | |
| --- | --- | --- | --- | --- | --- |
| **First order** | $r_{abtiotic}=k_{\mathrm{abtiotic}} {[Fe(II)][Fe(III)]\left[ \mathrm{NO}_{2}^{-} \right]}$ | NO_3_^-^ | 0.038 | NO_3_^-^ | 0.275 |
|  |  | NO_2_^-^ | 0.034 | NO_2_^-^ | 0.069 |
|  |  | Fe(II) | 0.069 | Fe(II) | 0.919 |
|  | $r_{abtiotic}=k_{\mathrm{abtiotic}} {[Fe(II)]\left[ \mathrm{NO}_{2}^{-} \right]}$ | NO_3_^-^ | 0.032 | NO_3_^-^ | 0.325 |
|  |  | NO_2_^-^ | 0.032 | NO_2_^-^ | 0.115 |
|  |  | Fe(II) | 0.068 | Fe(II) | 0.720 |
|  | $r_{abtiotic}=k_{\mathrm{abtiotic}} {[Fe(III)]\left[ \mathrm{NO}_{2}^{-} \right]}$ | NO_3_^-^ | 0.040 | NO_3_^-^ | 0.281 |
|  |  | NO_2_^-^ | 0.041 | NO_2_^-^ | 0.159 |
|  |  | Fe(II) | 0.070 | Fe(II) | 0.992 |
| **Multi-molecular** | $r_{abtiotic}=k_{\mathrm{abtiotic}} \left[ Fe(II) \right]^{4}\left[ Fe(III) \right]^{4} \left[ \mathrm{NO}_{3}^{-} \right]^{2}$ | NO_3_^-^ | 0.044 | NO_3_^-^ | 0.289 |
|  |  | NO_2_^-^ | 0.043 | NO_2_^-^ | 0.102 |
|  |  | Fe(II) | 0.075 | Fe(II) | 1.035 |
|  | $r_{abtiotic}=k_{\mathrm{abtiotic}} \left[ Fe(II) \right]^{4} \left[ \mathrm{NO}_{3}^{-} \right]^{2}$ | NO_3_^-^ | 0.032 | NO_3_^-^ | 0.446 |
|  |  | NO_2_^-^ | 0.049 | NO_2_^-^ | 0.284 |
|  |  | Fe(II) | 0.072 | Fe(II) | 0.526 |
|  | $r_{abtiotic}=k_{\mathrm{abtiotic}} \left[ Fe(III) \right]^{4} \left[ \mathrm{NO}_{3}^{-} \right]^{2}$ | NO_3_^-^ | 0.048 | NO_3_^-^ | 0.400 |
|  |  | NO_2_^-^ | 0.088 | NO_2_^-^ | 0.540 |
|  |  | Fe(II) | 0.085 | Fe(II) | 1.257 |

**References:**

Belli, K. M., DiChristina, T. J., Van Cappellen, P., & Taillefert, M. (2015). ‘Effects of aqueous uranyl speciation on the kinetics of microbial uranium reduction’, *Geochimica et Cosmochimica Acta*, 157: 109–24. DOI: 10.1016/j.gca.2015.02.006

Coby, A. J., & Picardal, F. W. (2005). ‘Inhibition of NO_3_^−^ and NO_2_^−^ Reduction by Microbial Fe(III) Reduction: Evidence of a Reaction between NO_2_^−^ and Cell Surface-Bound Fe^2+^’, *Applied and Environmental Microbiology*, 71/9: 5267–74. American Society for Microbiology. DOI: 10.1128/AEM.71.9.5267-5274.2005

Jones, L. C., Peters, B., Lezama Pacheco, J. S., Casciotti, K. L., & Fendorf, S. (2015). ‘Stable Isotopes and Iron Oxide Mineral Products as Markers of Chemodenitrification.’, *Environmental Science & Technology*, 49/6: 3444–52. American Chemical Society. DOI: 10.1021/es504862x

Kalyuzhnyi, S., Fedorovich, V., Lens, P., Hulshoff Pol, L., & Lettinga, G. (1998). ‘Mathematical modelling as a tool to study population dynamics between sulfate reducing and methanogenic bacteria’, *Biodegradation*, 9/3: 187–99. DOI: 10.1023/A:1008339018423

Poinapen, J., & Ekama, G. (2010). ‘Biological sulphate reduction with primary sewage sludge in an upflow anaerobic sludge bed reactor – Part 6: Development of a kinetic model for BSR’, 36/3.

Robles, Á., Vinardell, S., Serralta, J., Bernet, N., Lens, P. N. L., Steyer, J.-P., & Astals, S. (2020). ‘Anaerobic treatment of sulfate-rich wastewaters: process modeling and control’., pp. 277–317. DOI: 10.2166/9781789060959_0277
